# Cytokine screening identifies TNF to potentially enhance immunogenicity of pediatric sarcomas

**DOI:** 10.3389/fimmu.2024.1347404

**Published:** 2024-12-11

**Authors:** Hendrik Gassmann, Melanie Thiede, Jennifer Weiß, Emilie Biele, Luisa Flohé, Helena Lachermaier, Carolin Prexler, Valentina Evdokimova, Laszlo Radvanyi, Irfan Akhtar, Mina N. F. Morcos, Franziska Auer, Sebastian J. Schober, Julia Hauer, Uwe Thiel, Kristina von Heyking

**Affiliations:** ^1^ Department of Pediatrics, Children’s Cancer Research Center, Kinderklinik München Schwabing, TUM School of Medicine, Technical University of Munich, Munich, Germany; ^2^ Cancer Consortium (DKTK), partner site Munich, a partnership between DKFZ and University TUM School of Medicine, Technical University of Munich, Munich, Germany; ^3^ Ontario Institute for Cancer Research, Toronto, ON, Canada

**Keywords:** pediatric sarcomas, osteosarcoma, Ewing sarcoma, rhabdomyosarcoma, immunotherapy, adoptive T cell transfer, MHC-I, TNF

## Abstract

**Introduction:**

Pediatric sarcomas, including osteosarcoma (OS), Ewing sarcoma (EwS) and rhabdomyosarcoma (RMS) carry low somatic mutational burden and low MHC-I expression, posing a challenge for T cell therapies. Our previous study showed that mediators of monocyte maturation sensitized the EwS cell line A673 to lysis by HLA-A*02:01/CHM1^319^-specific allorestricted T cell receptor (TCR) transgenic CD8^+^ T cells (CHM1^319^ CD8^+^ T cells).

**Methods:**

In this study, we tested a panel of monocyte maturation cytokines for their ability to upregulate immunogenic cell surface markers on OS, EwS and RMS cell lines, using flow cytometry. xCELLigence, SRB and ELISpot assays were used to assess whether TNF pretreatment increases CD8^+^ T cell cytotoxicity.

**Results:**

We observed that TNF and IL-1β upregulated MHC class I, ICAM-1 as well as CD83 and PD-L1 on the surface of pediatric sarcoma cell lines, while IL-4, GM-CSF, IL-6 and PGE_2_ failed to induce respective effects. Although pretreatment of pediatric sarcoma cell lines with TNF did not improve unspecific peripheral blood mononuclear cells (PBMCs) cytotoxicity, TNF enhanced specific lysis of 1/3 HLA-A2^+^ EwS cell lines by CHM1^319^ CD8^+^ T cells depending on MHC-I expression and ICAM-1 upregulation.

**Discussion:**

Our study supports utilization of TNF or TNF-inducing regimens for upregulation of MHC-I and costimulatory surface molecules on pediatric sarcoma cells and for enhancing recognition of responsive HLA-A2^+^ EwS tumor cells by antigen-specific CD8^+^ T cells.

## 1 Introduction

Pediatric sarcomas account for 10% of all childhood cancers, of which osteosarcoma (OS) and Ewing sarcoma (EwS) are the two most frequent bone cancers, while rhabdomyosarcoma (RMS) is the most common soft tissue tumor (1). Pediatric sarcomas are considered non-immunogenic tumors with low mutational burden (2), low MHC-I expression (3–6) and scarce T cell infiltration (7–9). Thus far immune checkpoint blockade failed to show clinical benefit in pediatric sarcomas (9–12). Therefore, immunogenic conditioning of pediatric sarcomas prior to T cell mediated immunotherapy is of particular importance.

We previously generated HLA-A*02:01/CHM1^319^-specific allorestricted CD8^+^ T cells (CHM1^319^ CD8^+^ T cells) targeting EwS, which exerted anti-tumoral effects *in vitro* and *in vivo*. Furthermore, CHM1^319^ CD8^+^ T cells were associated with a partial response in at least one out of three treatment-refractory EwS patients (13–15). We also observed that the pretreatment of the EwS cell line A673 with mediators of monocyte cell maturation, including GM-CSF and IL-4 for 5 days followed by, IL-1β, IL-6, TNF and PGE_2_ for 2 days, enhanced the recognition and cytotoxicity of CHM1^319^ CD8^+^ T cells against A673 cells *in vitro* (16). Yet, for the clinical setting, the administration or induction of six different cytokines is impracticable.

In this study, we hypothesized that individual monocyte maturation mediators induce immunogenicity of pediatric sarcoma cells, thereby enhancing their recognition and killing by healthy donor peripheral blood mononuclear cells (PBMCs) or CHM1^319^ CD8^+^ T cells. Among six cytokines tested, we identified TNF as the most prominent MHC-I and ICAM-1 inducer and CHM1^319^ CD8^+^ T cell sensitizer. As such, our study provides the basis for further evaluating TNF or TNF-inducing conditioning regimes prior to adoptive T cell therapies in pediatric sarcomas.

## 2 Materials and methods

### 2.1 Cell lines and cell culture conditions

OS (SJSA-1, U2OS), EwS cell lines (A673, CHLA10, EW7, TC32, TC71, TTC466, TTC633, SB-KMS-KS1/SBSR-AKS and SKNMC) and RMS cell lines (RD, RH30) were used in this study. Cells were purchased from American Type Culture Collection (ATCC): A673, MRC5 fibroblasts, RD, SJSA-1 and U2OS, or the German Collection of Microorganisms and Cell Cultures (DSMZ): RH30, SKNMC and TC71. SB-KMS-KS1 was established at the Children’s Cancer Research Center, Kinderklinik Schwabing (Technical University Munich, Germany). CHLA10, TC32, TTC466 and TTC633 were kindly provided by Prof. Poul Sorensen (University of British Columbia, Canada), which were originally obtained from the Childhood Cancer Repository (CCR, Alex’s Lemonade Stand Foundation, Children’s Oncology Group, COG). EW7 was kindly provided by Prof. Olivier Delattre (Institut Curie, Paris, France). OS cell lines, EwS cell lines and RH30 were cultured in RPMI 1640 medium (Life Technologies Limited, Paisley, UK). RD was cultured in DMEM (Life Technologies Limited, Paisley, UK), and CHLA10 was cultured in Iscove’s Modified Dulbecco’s Medium (IMDM) supplemented with 4 mM L-Glutamine and 1X ITS (5 μg/ml insulin, 5 μg/ml transferrin, 5 ng/ml selenous acid). MRC5 and TTC633 were cultured in DMEM/F12 (Life Technologies Limited Paisley, UK). Growth medium of all cell lines was supplemented with 10% fetal bovine serum (FBS) (Life Technologies Limited, Paisley, UK), 100 U/ml penicillin and 100 µg/ml streptomycin (Life Technologies Corporation, Grand Island, NY, USA). All cell lines were cultured at 37°C with 5% CO_2_ and regularly tested for mycoplasma contamination (Lonza).

For cytokine treatment, 0.005 –2 x 10^5^ pediatric sarcoma cells were seeded in flat bottom 6-well plates (Falcon) or 96-well plates (Sartorius, Germany) in 3 ml or 200 µl of growth medium, respectively. Next day, the medium was replaced and supplemented with either 800 U/ml GM-CSF (Sanofi, Paris, France), 1000 U/ml IL-4, 10 ng/ml IL-1β, 1000 U/ml IL-6, 1-100 ng/ml TNF or 1 µg/ml PGE_2_ (all from R&D Systems, Minneapolis, MN, USA). As a positive control, cells were treated with 100 U/ml IFNγ (R&D Systems, Minneapolis, MN, USA). Cells were treated for 72 h or 96 h and analyzed by flow cytometry. For western blotting, cells were treated with 10 ng/ml IL-1β (#IL038, Millipore), 10 ng/ml IL-6 (#GF338, Millipore) or 10 ng/ml TNF (#PHC3011, Thermo Fisher), or 10 ng/ml IFNα (#8927, Cell Signaling) as positive control. Where indicated, EwS cells were preincubated with monocyte maturation mediators (DC cytokines), as described previously for the generation of monocytic-derived dendritic cells (16, 17). Briefly, EwS cells were incubated with 800 U/ml GM-CSF and 1000 U/ml IL-4 for 5 days, with medium renewal at day 3. At day 6, fresh medium supplemented with IL-1β, IL-6, TNF (10 ng/ml) and PGE_2_ (concentrations as above) was added, and cells were harvested for analysis at day 7.

### 2.2 PBMC isolation

The study was conducted in accordance with the Declaration of Helsinki and approved by the Ethics Commission of the Medical Faculty of the Technical University of Munich (2562/09). Healthy donor buffy coats were acquired from the DRK-Blutspendedienst (Ulm, Germany) or, after informed consent, venous EDTA-blood was obtained from healthy donors. Density-gradient centrifugation with Ficoll-Paque (GE Healthcare, Uppsala, Sweden) was used to isolate PBMCs as reported previously (18). The ACK Lysis Buffer (Thermo Fisher Scientific) was used to remove contaminating erythrocytes.

### 2.3 Generation of HLA-A*02:01/CHM1^319^-specific allorestricted T-cell receptor transgenic CD8^+^ T cells

HLA-A*02:01/CHM1^319^-specific allorestricted T-cell receptor (TCR) transgenic CD8^+^ T cells (CHM1^319^ CD8^+^ T cells) were generated as described previously (14, 18, 19). Briefly, CD8^+^ T cells were negatively isolated (untouched, Miltenyi Biotech, Germany) from healthy donor PBMCs and activated with anti-CD3/CD28 Dynabeads™ (Thermo Fisher Scientific BalticsUAB, Vilnius, Lithuania) according to the supplier’s instructions and 100 U/ml recombinant human (rh) IL-2 (Novartis) for 2 days. At day 3 and 4, T cells were retrovirally transduced with the CHM1^319^ TCR transgene using the retroviral vector pMP-71 (14). Transduction efficacy was evaluated by flow cytometry and T cells were selected using anti-PE microbeads (Miltenyi Biotech, Germany) bound to PE anti-mouse TCRβ chain antibody (H57-587, BioLegend) recognizing the murinized constant region of the CHM1^319^ CD8^+^ T cells. T cells were cultured in AIM-V medium (Life Technologies Limited, Paisley, UK) supplemented with 5% human AB serum (Sigma-Aldrich, Germany), 100 U/ml IL-2, 2 ng/ml IL15 (R&D Systems) and antibiotics, as described above.

### 2.4 Flow cytometry

To assess the surface expression of immunogenic markers, 0.5-2 x 10^5^ cells were stained in 96-well plates in 50 µl PBS with 1:100 diluted fluorochrome-conjugated antibodies against CD86 (REA968, VioBlue), CD83 (REA714, APC-Vio770), PD-L1 (REA1197, VioBright FITC), MHC-I (HLA-A,B,C; REA230, APC), MHC-II (HLA-DR, DP, DQ; REA332, PE-Vio770), PD-1 (REA1165, APC), CD152/CTLA-4 (BNI3, APC), CD223/LAG-3 (REA351, APC), CD366/TIM-3 (REA635, APC) or respective isotype controls (mouse IgG2a-FITC, mouse IgG1-APC/IS5-21F5 or REA293), at 4°C protected from light. All antibodies were obtained from Miltenyi Biotech, Germany, except for HLA-A2 (BB7.2) and CD152/CTLA-4 (BNI3), both BD Biosciences. Transduction of CHM1^319^ CD8^+^ T cells was confirmed by staining the murinized part of the TCR with an anti-mouse β TCR chain antibody (H57-597, PE), obtained from BioLegend. After 15 min-incubation, cells were washed with 200 µl PBS at 300 g for 5 min, resuspended in 100 µl PBS and acquired on the MACSQuant Analyzer 10 (Miltenyi Biotech, Germany). Single stainings of respective cells were used for compensation of multicolor staining panels. Data were analyzed using FlowJo™ 10.1 (BD Bioscience, Heidelberg, Germany) and applying the following gating strategy (1): doublet discrimination (FSC-H vs. FSC-A) (2), gating on cells (SSC-A vs. FSC-A) (3), dead cell exclusion using PI (B3 vs. B2; **Supplementary Figure 1**).

### 2.5 Western blotting

To quantify the total protein expression of immunogenic markers, cells were lysed on the plates with RIPA buffer (#20-188, Millipore) supplemented with protease inhibitor cocktail (Roche Diagnostics). Western blotting was performed as described previously (20). In brief, cell extracts were normalized for protein concentration using the Pierce BCA Protein Assay kit (#23225; Thermo Fisher), diluted 5 times in Laemmli buffer, boiled and resolved by sodium dodecyl sulfate (SDS) gel electrophoresis in 10% polyacrylamide gels. After transferring to 0.45 µm nitrocellulose membranes (#1620115; Bio-Rad), the membranes were blocked for 1 h with 5% milk-TBST (20 mM Tris-HCl, pH 7.8, 150 mM NaCl, 0.1% Tween-20) and incubated overnight with primary antibodies diluted at 1:1,000 in 0.5% milk-TBST. The membranes were then washed three times with TBST, followed by the 1 h incubation with the secondary peroxidase-linked anti-mouse or anti-rabbit IgGs (Cell Signaling) diluted at 1:5,000 in 0.5% milk-TBST. After washing the membrane with TBST, the chemiluminescent signals were detected with the ECL solution (RPN2232; Amersham) using the ChemiDoc XRS+ imaging instrument. The primary antibody against MHC-I (W6/32, sc-32235), was obtained from Santa Cruz Biotechnology. GAPDH (ab9485) was purchased from Abcam.

### 2.6 xCELLigence real-time cell growth assay

For the xCELLigence growth assay, 1 x 10^4^ TNF-pretreated cells (A673, TC32, SJSA-1, U2OS, RD and RH30) were seeded in 150 µl medium/well in 96-well E-plates (Roche Diagnostics, Penzberg, Germany). To measure cytotoxic activity of PBMCs, freshly isolated healthy donor-derived PBMCs were added 24 h or 48 h later, at effector to target ratios (E:T) of 0.5:1, 1:1, 2:1, 5:1 or 10:1, to a final volume of 250 µl/well. Real-time cell growth was monitored in 15 min intervals for a maximum of 120 h using the impedance-based xCELLigence RTCA SP system (Roche Diagnostics, Penzberg, Germany).

### 2.7 Sulforhodamine B assay

SRB assay was performed as described previously (21). Briefly, 1 x 10^4^ TNF-pretreated EwS cells were seeded in 96-well plates, in a total volume of 150 µl/well. Next day, CHM1^319^ CD8^+^ T cells were added at an E:T ratio of 1:1 or as indicated in the figure legends. After 24 h incubation, the supernatant was collected, centrifuged at 300 g for 5 min to remove cells and stored at -80°C. Cells were fixated with ice-cold 10% trichloroacetic acid overnight at 4°C and stained with 0.5% SRB (Sigma-Aldrich) in 1% acetic acid for 30 min. The cytotoxic effect was quantified by solubilizing the cell-bound SRB in 10 mM Tris buffer (pH 10) and measuring the optical density at 510 nm using the absorbance plate reader (Infinite M Nano, Tecan).

### 2.8 ELISpot assay

96-well mixed cellulose ester plates (MultiScreen-HA Filter Plate, 0,45 μm, Millipore, Germany) were coated with 10 μg/ml IFNγ capture antibody (mAB 1-D1K) or 10 µg/ml Granzyme B capture antibody (mAB GB10) in 50 µl PBS/well at 4°C over night. Next day, plates were washed 4x with 200 μl PBS for 10 min each at 4°C, and then blocked with 150 μl of T cell medium/well at 37°C for 1 h. Unspecific or CHM1^319^ CD8^+^ T cells were seeded onto the coated 96-well mixed cellulose ester plates (5,000-20,000 cells/well) in 50 µl T cell medium/well. After 30 min, 20,000 EwS cells in 50 µl T cell medium/well were carefully added. For MHC-I and ICAM-1 blockade experiments, EwS cells were preincubated either with 1-100 µg/ml W6/32 antibody (Helmholtz Zentrum München, Germany), or an anti-ICAM-1 antibody (0.1-20 µg/ml; clone P2A4, MAB2146Z, Millipore) or IgG1 control (20 µg/ml; CBL610; Millipore) at 37°C for 30 min. For positive and negative controls, 1 x 10^7^ T2 cells/ml were pulsed with 35 µg/ml of a CHM1^319^ or Influenza peptide (GILGFVFTL; both Thermo Fisher) at 37°C for 2 h, washed and added at a concentration of 20,000 cells/50 µl/well (22). Cells were co-cultured for 20 h at 37°C. Next day, plates were washed 6x with 0.05% Tween (Sigma) in PBS and dried briefly. The wells were incubated with 2 μg/ml IFNγ detection antibody (7B61-Biotin) or 2µg/ml Granzyme B detection antibody (GB11-Biotin) in 0.5% BSA-PBS/well at 37°C for 2 h. After washing 6x with 0.05% Tween in PBS, plates were dried and incubated with 100 μl of Streptavidin-HRP at a 1:100 dilution in 0.5% BSA-PBS at room temperature and protected from light for 1 h. Plates were washed 3x with 0.05% Tween in PBS, 3x with PBS, dried briefly and developed using developing solution freshly prepared by adding 25 μl of 30% (w/w) H_2_O_2_ solution (Sigma) per 10 ml of 3-Amino-9-ethylcarbazole solution (Sigma); 100 μl/well. Plates were incubated for up to 8 min, washed with running tap water and air dried thoroughly. Spots were counted using the ELISpot reader (AID iSpot, light source AID Xenon Light Source ELR0024, AID Autoimmun Diagnostika GmbH).

### 2.9 Quantitative RT-PCR

Total RNA of A673 and TC32 EwS cell lines was isolated using the RNeasy Plus Mini Kit (Quiagen) after three days of incubation with 0, 1, 10 or 100 ng/ml TNF and reverse transcribed using the High Capacity cDNA Reverse Transcription Kit (Thermo Fisher Scientific) according to the manufacturer’s instructions. Differential gene expression of CHM1 (HS00170877_m1) was then analyzed by qRT-PCR using TaqMan Universal PCR Master Mix and fluorescence detection with a QuantStudio6Pro (Applied Biosystems). Gene expression was normalized to glyceraldehyde-3- phosphate dehydrogenase (GAPDH; Hs99999905_m1) using the ΔΔCT method (all Applied Biosystems).

### 2.10 Statistical analysis

Statistical analyses were performed and graphs plotted using GrapPadPrism 9 (GraphPad Software, San Diego, CA, USA). One-way or two-way ANOVA with multiple comparison Tukey test, Dunnett´s test or Šidák test were performed to calculate p values. ns not significant, * p ≤ 0.05, ** p ≤ 0.01, *** p ≤ 0.001, **** p ≤ 0.0001. The illustrations were created with Biorender.com.

## 3 Results

### 3.1 TNF and IL-1β upregulate immunogenic surface markers on pediatric sarcoma cell lines

**Figure 1:**
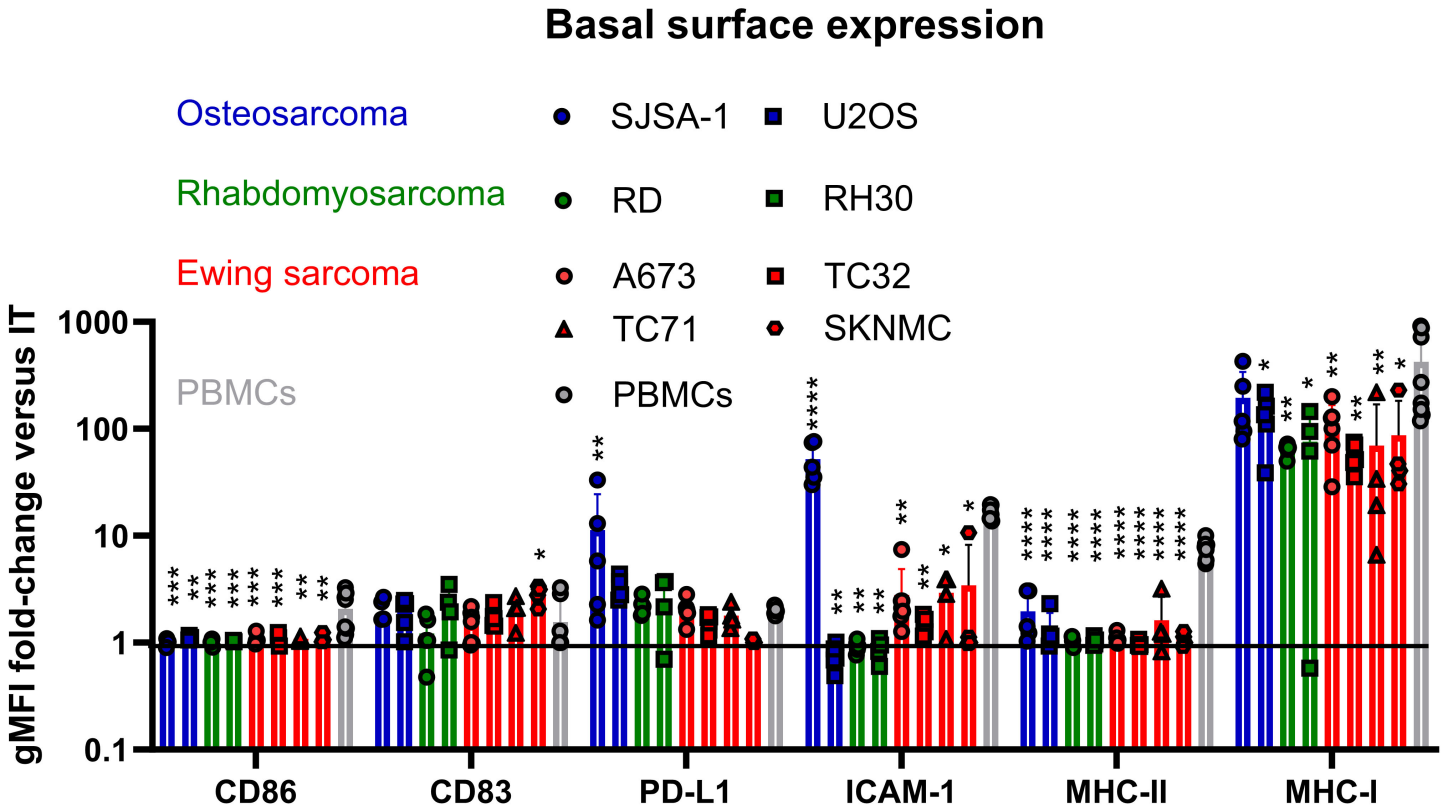
Pediatric sarcomas exhibit lower MHC-I expression and lack co-stimulatory surface molecules compared to healthy donor PBMCs. Flow cytometry of osteosarcoma (SJSA-1, U2OS), rhabdomyosarcoma (RD, RH30) and Ewing sarcoma (A673, TC32, TC71 and SKNMC) cell lines compared to healthy donor PBMCs. Surface expression is displayed as ratio of the geometric mean fluorescence intensities (gFMI) of the specific staining and isotype control. Ratio of 1 indicates lack of surface expression. Each symbol represents an independent experiment or donor with at least two replicates. Data are presented as mean ± SD. One-way ANOVA with multiple comparison Dunnett`s test was used to calculate p values. * p ≤ 0.05, ** p ≤ 0.01, *** p ≤ 0.001, **** p ≤ 0.0001.

We assessed a panel of pediatric sarcoma cell lines, including OS (SJSA-1, U2OS), RMS (RD, RH30) and EwS (A673, TC32, TC71 and SKNMC) for their basal expression of the immunogenic surface markers CD86, CD83, PD-L1, ICAM-1, MHC-I and MHC-II in comparison to healthy donor-derived PBMCs. Flow cytometry showed a variable expression of MHC-I on all tested cell lines, with the highest levels detected in the OS cell lines SJSA-1 and U2OS as well as the EwS cell line A673, and the lowest in TC71 and SKNMC (**Figure 1**, **Supplementary Figure 2A**). Compared to healthy donor PBMCs, MHC-I was significantly lower on all pediatric sarcoma cell lines, except for the OS cell line SJSA (**Figure 1**, **Supplementary Figure 2B**). Notably, SJSA-1 and U2OS cells showed PD-L1 expression (**Figure 1**, **Supplementary Figure 2A**), demonstrating a mixed expression of immunogenic and immunosuppressive markers among pediatric sarcomas; which is in accordance with partial PD-L1 expression in osteosarcomas (5, 23, 24). SJSA-1 was the only pediatric sarcoma cell line with basal ICAM-1 surface expression. None of the tested cell lines was positive for CD83, CD86 or MHC-II, which is in line with previous observations in EwS (3).

**Figure 2:**
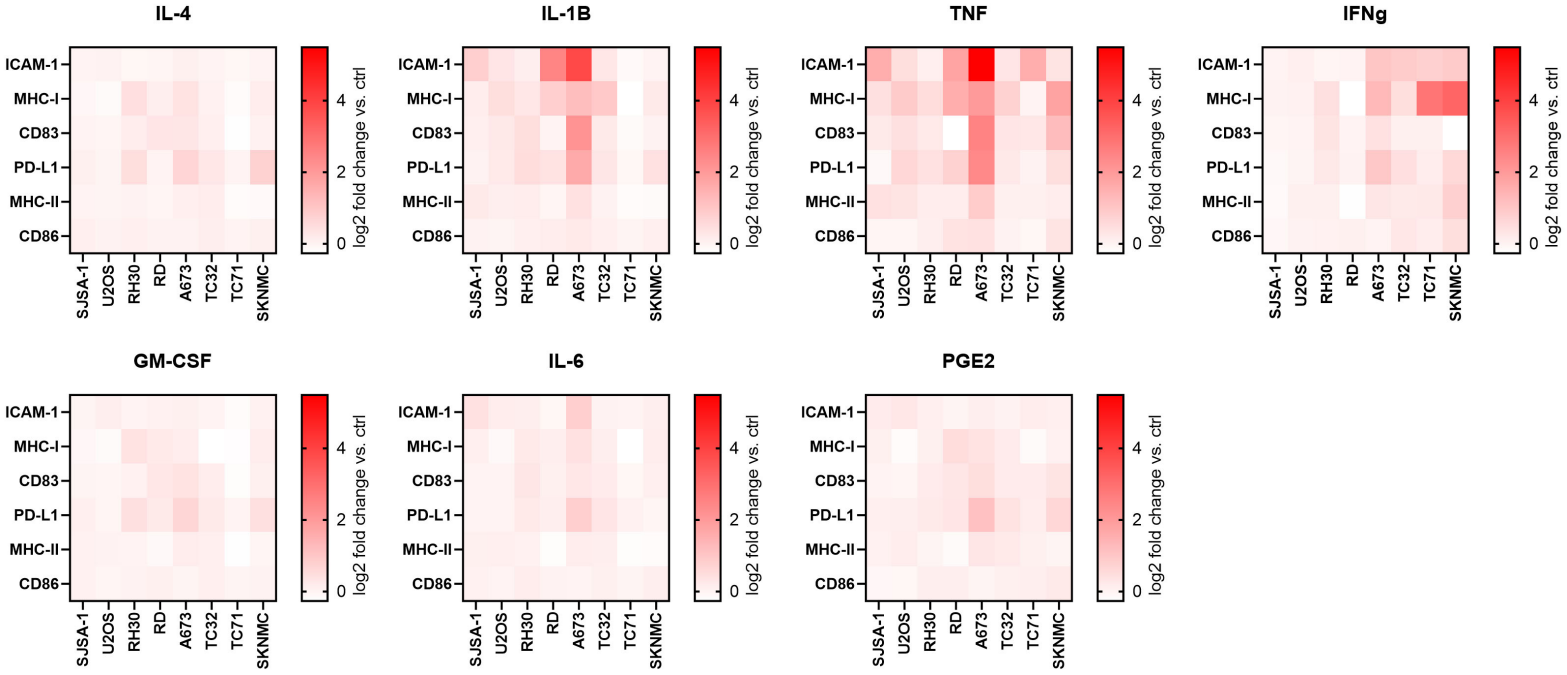
TNF and IL-1β upregulate immunogenic surface markers on pediatric sarcoma cell lines. Flow cytometry of eight pediatric sarcoma cell lines after 96 h treatment with indicated cytokines or left untreated (ctrl). IFNγ-treatment was used as positive ctrl. Shown are heatmaps for the log2 fold change of the geometric mean fluorescence intensities (gMFI) of the specific staining minus gMFI of the respective isotype ctrl of the cytokine-treated versus ctrl cells. Results from two (SJSA-1, U2OS, RH30, RD, A673 and TC32) or three (TC71, SKNMC) independent experiments with three replicates each are shown.

To test if the surface expression of immunogenic markers can be enhanced, pediatric sarcoma cell lines were incubated for 96 h with individual cytokines commonly used together for the differentiation and maturation of monocytic-derived dendritic cells (DC), including IL-4, GM-CSF, IL-1β, IL-6, TNF and PGE_2_ (13, 16). As these cytokines exert pleiotropic and context-dependent functions, we tested their individual effect on pediatric sarcoma cells in an unbiased manner. Flow cytometry analyses showed that IL-1β and TNF were the most efficient inducers of ICAM-1 and MHC-I in the majority of cell lines, while IL-4, GM-CSF, IL-6 and PGE_2_ had weak to no effects (**Figure 2**, **Supplementary Figures 3** and **4A**). The effects of IL-1β and TNF were partly comparable to the positive control IFNγ, which strongly induced MHC-I on TC71 and SKNMC, and slightly upregulated ICAM-1 on A673, TC32, TC71 and SKNMC, which is in line with previous reports (25). Western blots confirmed that IL-1β, TNF and IFNα (used as positive control) were inducers of MHC-I in A673 and TC32 (**Supplementary Figure 4B**), with TNF showing the strongest MHC-I stimulation in A673. In some cell lines (most notably, A673), IL-1β and TNF also induced CD83 (but not CD86 or MHC-II) and PD-L1. In RMS RD cells, IL-1β induced higher ICAM-1 expression than TNF (**Supplementary Figures 5**, **6**). Overall, A673 cells were the strongest responders and TNF was the most potent inducer of immunogenic surface markers. These markers, including ICAM-1, MHC-I and CD83 were variably induced by TNF in the pediatric sarcoma cell lines tested here.

**Figure 3:**
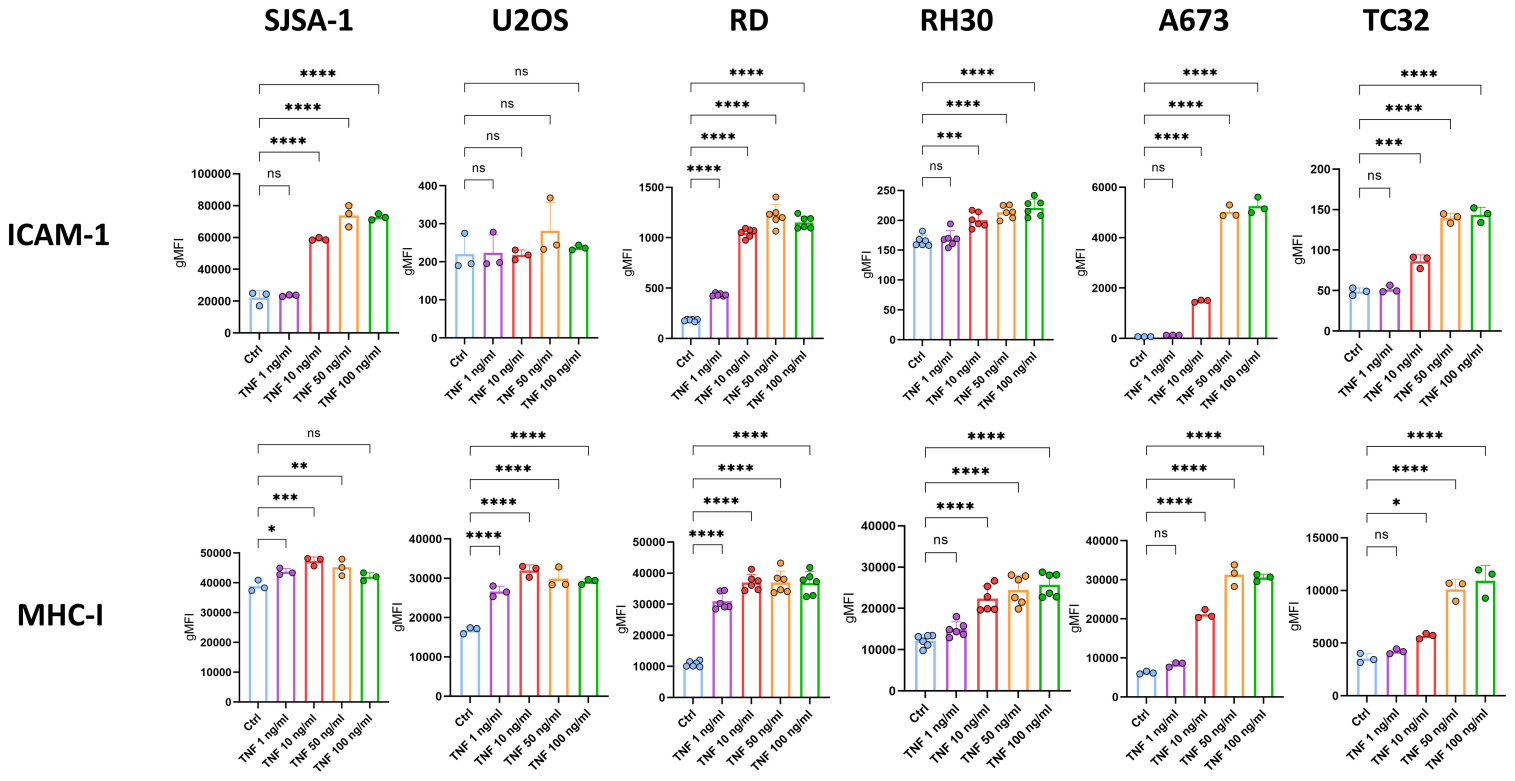
TNF dose-dependently upregulates ICAM-1 and MHC-I on pediatric sarcoma cell lines. Flow cytometry of pediatric sarcoma cell lines after 96 h treatment with indicated doses of TNF or left untreated as control (ctrl). Expression of ICAM-1 and MHC-I is shown as geometric mean fluorescence intensities (gMFI) minus gMFI of the respective isotype ctrl. Representative results from three independent experiments with three (SJSA-1, U2OS, A673, TC32) or six (RD, RH30) replicates are shown. Data are presented as mean ± SD. One-way ANOVA with multiple comparison Dunnett´s test was used to calculate p values. ns = not significant, * p ≤ 0.05, ** p ≤ 0.01, *** p ≤ 0.001, **** p ≤ 0.001.

Importantly, TNF-mediated upregulation of MHC-I and ICAM-1 on RD, SJSA-1 and A673 was dose-dependent (**Figure 3**), suggesting physiological relevance. However, their upregulation was less prominent on RH30 and TC32 (≤ 2-fold change *vs* control), and U2OS showed no change for ICAM-1 (**Figure 3**). In some cell lines, including U2OS, RH30 and A673, TNF also slightly upregulated CD83 as well as PD-L1 (**Supplementary Figures 7**-**9**). Using the additional EwS cell lines CHLA10 and EW7, we confirmed the dose-dependent upregulation of both MHC-I and ICAM-1 in response to TNF, although ICAM-1 upregulation was weaker compared to A673 cells (**Supplementary Figure 9**).

Due to the response to TNF, surface expression of its two receptors TNFR1 and TNFR2 was evaluated on pediatric sarcoma cell lines using flow cytometry. All pediatric sarcoma cell lines tested showed modest to strong TNFR1 expression, while TNFR2 was weakly expressed. RD was the only cell line with strong TNFR2 expression, at similar levels compared to healthy donor PBMCs (**Supplementary Figure 10**).

**Figure 4:**
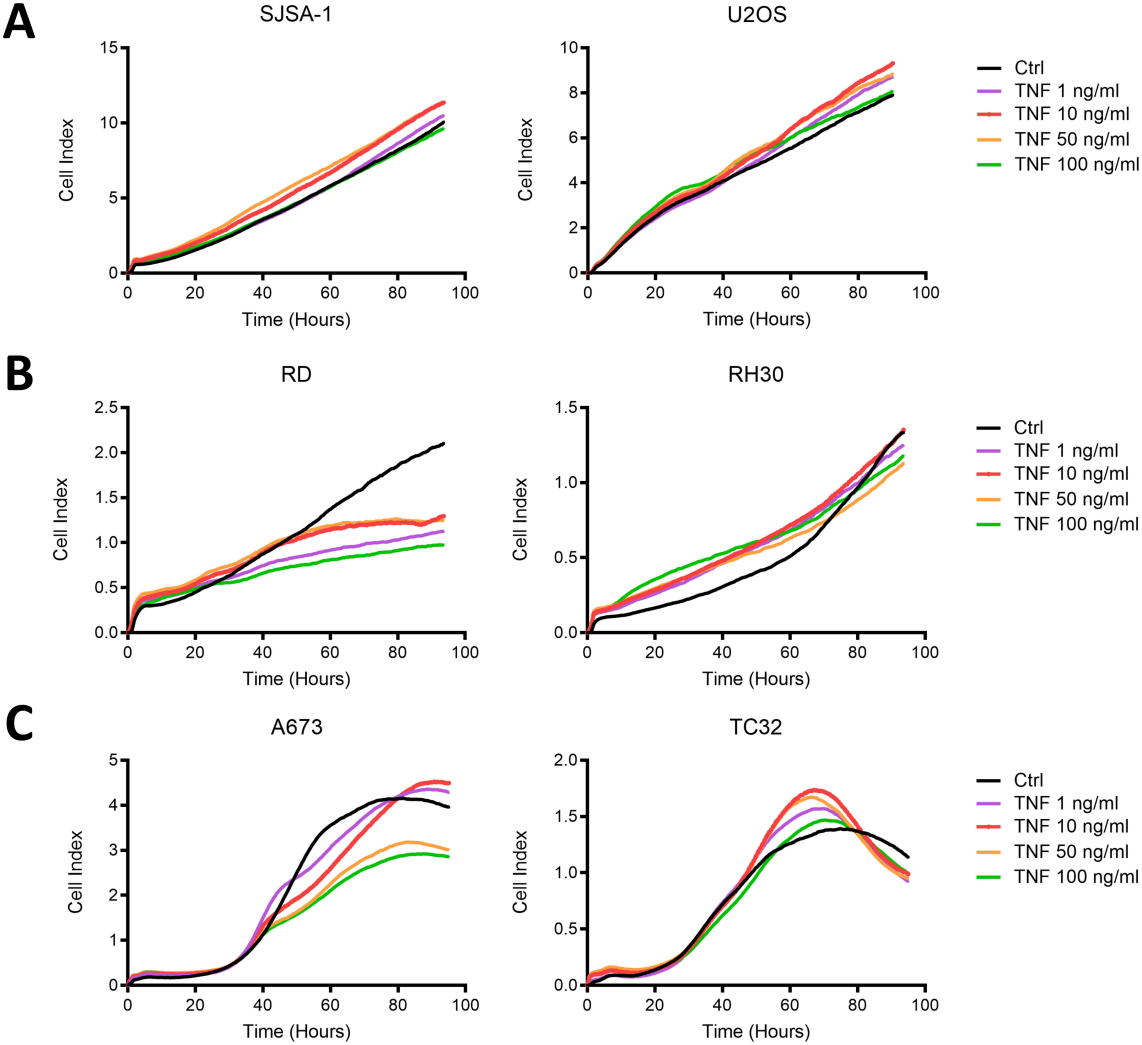
TNF inhibits the growth of RMS cell line RD and EwS cell line A673, but not OS cell lines or EwS TC32 *in vitro*. Live cell growth assay xCELLigence of **(A)** osteosarcoma (OS) cell lines, **(B)** rhabdomyosarcoma (RMS) cell lines or **(C)** Ewing sarcoma (EwS) cell lines in the presence of indicated doses of TNF. Representative results from two (U2OS, SJSA-1, A673, TC32) or three (RD, RH30) independent experiments with three replicates are shown.

Associated with the strong upregulation of immunogenic surface markers on RD and A673 cells, TNF inhibited their growth, while RH30, TC32 and the OS cell lines SJSA-1 and U2OS were not affected (**Figure 4**). Therefore, TNF is capable of upregulating ICAM-1, MHC-I and, to a lesser extent, CD83 and PD-L1 on multiple pediatric sarcoma cell lines without promoting tumor growth *in vitro*, and thus was further assessed for its immunostimulatory activity.

### 3.2 TNF sensitizes A673 cells to EwS antigen-specific CD8^+^ T cells depending on MHC-I and ICAM-1

**Figure 5:**
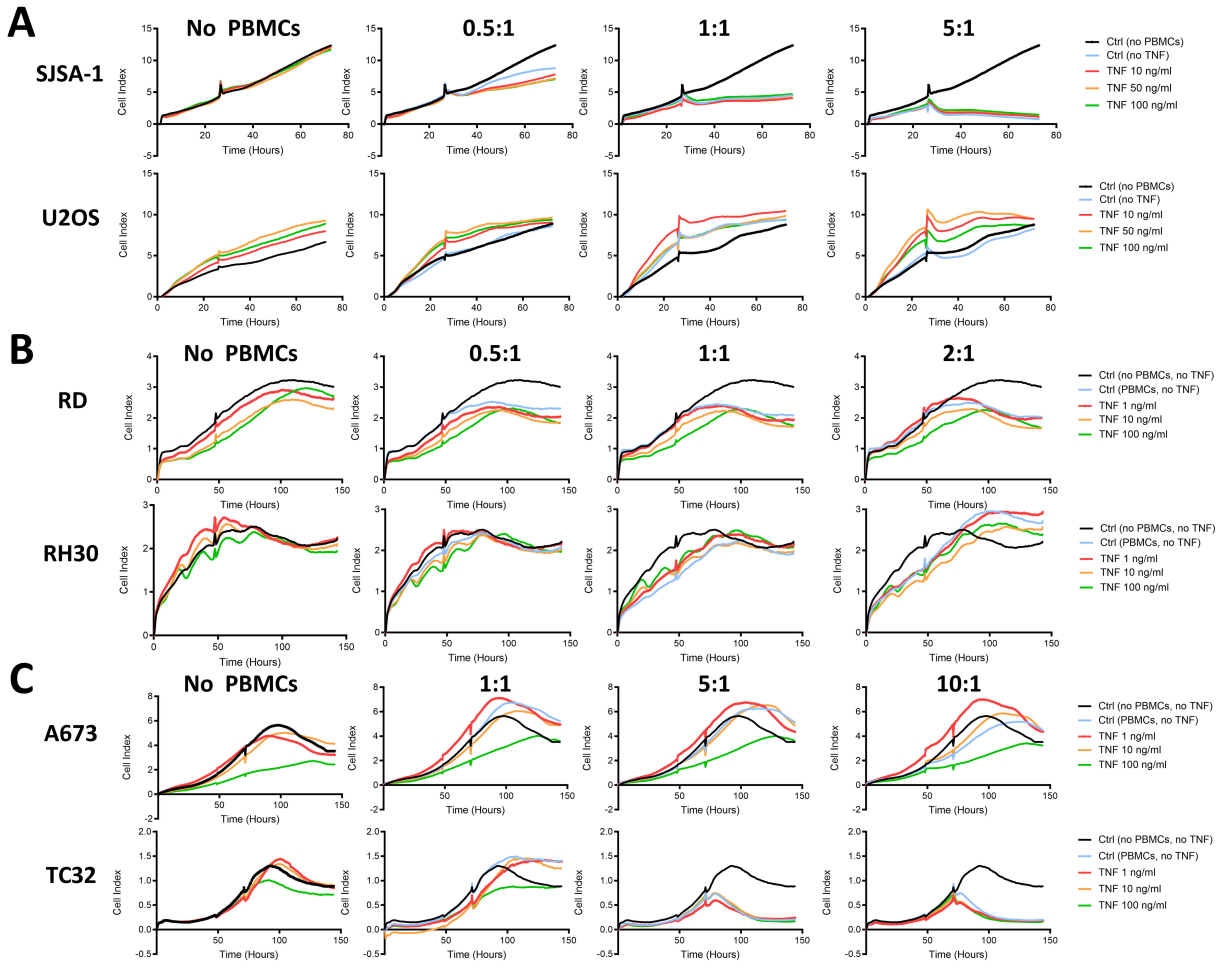
TNF-pretreatment of pediatric sarcoma cell lines fails to enhance cytotoxicity of HLA-mismatched PBMCs. Live cell growth assay xCELLigence of **(A)** osteosarcoma (SJSA-1, U2OS), **(B)** rhabdomyosarcoma (RD, RH30) and **(C)** Ewing sarcoma (A673, TC32) cell lines pretreated with indicated amount of TNF for 96 h prior to seeding. Healthy donor-derived, HLA-mismatched PBMCs were added at the indicated effector to target ratios to tumor cells after 48 h or when the cell index reached 1-2 (small notch represents addition of PBMCs). Representative results from three independent experiments with three independent donors in triplicates are shown.

As TNF induced expression of co-stimulatory molecules on OS, RMS and EwS cell lines (**Figures 2**, **3**), we tested whether pretreatment with TNF sensitizes pediatric sarcoma cell lines towards killing by HLA-mismatched healthy donor-derived PBMCs. Using the xCELLigence assay, we observed that HLA-mismatched PBMCs exerted partial tumor control against SJSA-1 at effector to target ratios (E:T) of 1:1 and 5:1 and against TC32 at E:T 5:1 and 10:1 (**Figures 5A, C**), but failed to control growth of U2OS, RD, RH30 and A673 (**Figures 5A–C**). However, pretreatment of these cell lines with a range of TNF concentrations did not improve the anti-tumor activity of HLA-mismatched PBMCs against these pediatric sarcoma cell lines (**Figure 5**), indicating that the upregulated co-stimulatory molecules by TNF do not promote the anti-tumor activity of unspecific PBMCs. This could be due to the resting state or the low antigen specificity of HLA-mismatched PBMCs.

**Figure 6:**
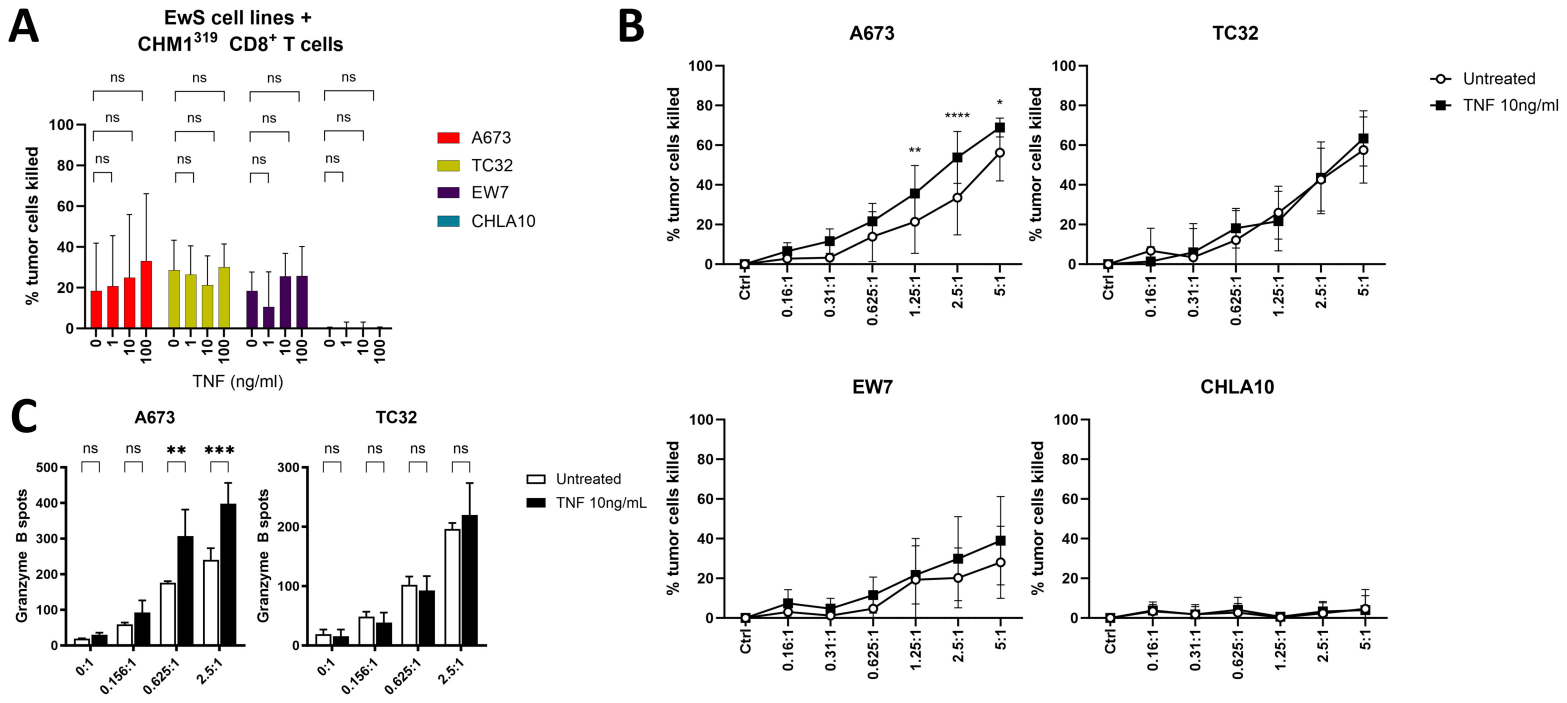
TNF-pretreatment partly sensitizes EwS cells against antigen-specific CD8^+^ T cells. **(A, B)** SRB assay showing cell growth of four EwS cell lines pretreated with different amounts of TNF or left untreated for 4 days. HLA-A*02:01/CHM1^319^-specific allorestricted TCR transgenic CD8^+^ T cells (CHM1^319^ CD8^+^ T cells) were added at an effector to target ratio of 1:1 **(A)** or as indicated **(B)** and analyzed after 24 h. **(C)** Granzyme B ELISpot of CHM1^319^ CD8^+^ T cells co-cultured at indicated effector to target rations with untreated or TNF-pretreated (10ng/ml, 72 h) A673 and TC32 for 24 h. **(A)** Combined results from three (EW7, CHLA10) or four (A673, TC32) independent experiments with three or four independent donors in quadruplicates are shown. Combined results from four **(B)** or representative results from three **(C)** independent experiments with four **(B)** or three **(C)** independent donors in triplicates are shown. Data are presented as mean ± SD. Two-way ANOVA with Dunnett´s **(A)**, Šídák´s **(B)** or Tukey´s **(C)** multiple comparisons test was used to calculate p values. ns = not significant, * p ≤ 0.05, ** p ≤ 0.01, *** p ≤ 0.001, **** p ≤ 0.0001.

Because TNF-pretreatment of pediatric sarcoma cell lines did not promote anti-tumor activity of unspecific PBMCs, we tested whether pretreatment with TNF sensitizes EwS cell lines to killing by antigen-specific CHM1^319^ CD8^+^ T cells. As shown by SRB assay, CHM1^319^ CD8^+^ T cells lysed HLA-A2^+^ (A673, TC32, EW7), but not HLA-A2^-^ (CHLA10) EwS cell lines (**Figure 6A**, **Supplementary Figure 11**), in line with our previous findings (26). The pretreatment of EwS cell lines with increasing doses of TNF did not further improve tumor cell lysis by CHM1^319^ CD8^+^ T cells at the E:T of 1:1 (**Figure 6A**), apart from A673, which showed a tendency to be sensitized by the increasing doses of TNF. However, at increased E:T of 1.25:1, 2.5:1 and 5:1, the pretreatment with 10 ng/ml TNF significantly improved killing of A673 by CHM1^319^ CD8^+^ T cells, while TC32 and EW7 cells remained unaffected (**Figure 6B**). The lysis of HLA-A2^-^ EwS cell line CHLA10 was not altered by pretreatment with TNF (**Figure 6B**), confirming specificity of CHM1^319^ CD8^+^ T cells towards HLA-A2-positive cells. Granzyme B ELISpot showed that TNF-pretreatment of A673, but not TC32, increased Granzyme B release from CHM1^319^ CD8^+^ T cells at E:T of 0.625:1 and 2.5:1 (**Figure 6C**), confirming increased lysis of A673 after TNF-pretreatment.

To investigate the potential long-term effect of TNF pretreatment on CHM1^319^ CD8^+^ T cells, it was evaluated, if TNF-pretreatment of EwS cell lines promotes exhaustion of CHM1^319^ CD8^+^ T cells using flow cytometry. The exhaustion markers TIM-3, PD-1, CTLA-4 and LAG-3 were not significantly increased on CHM1^319^ CD8^+^ T cells after 96 h-coculture with TNF-pretreated compared to untreated A673 and TC32 cells (**Supplementary Figure 12**). PD-1 and LAG-3 were significantly upregulated on CHM1^319^ CD8^+^ T cells after co-culture with EwS cell lines in comparison to CHM1^319^ CD8^+^ T cells only, indicating the induction of exhaustion in CHM1^319^ CD8^+^ T cells after antigen stimulation by EwS cell lines independent of TNF-pretreatment. Overall, PD-1 and LAG-3 expression levels showed inter-donor heterogeneity (data not shown). Therefore, TNF-pretreatment of EwS cell lines A673 and TC32 does not enhance an exhaustion phenotype in antigen-specific CD8^+^ T cells *in vitro*.

**Figure 7:**
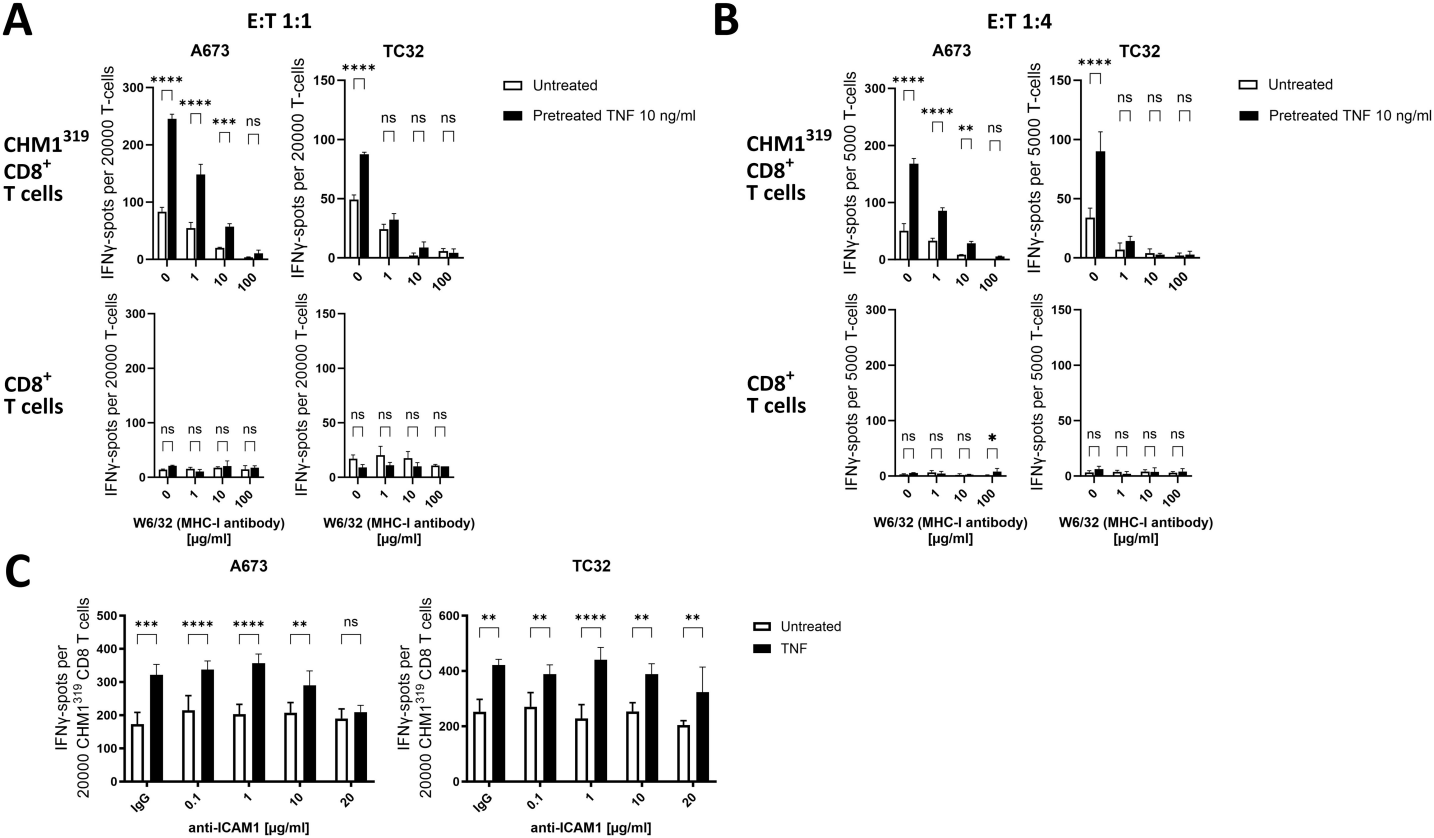
TNF-pretreatment sensitizes EwS cell lines to antigen-specific CD8^+^ T cells depending on MHC-I and ICAM-1 upregulation. **(A–C)** ELISpot assay showing count of IFNγ spots. Ewing sarcoma (EwS) cells A673 and TC32 were pretreated with 10ng/ml TNF for 72 h and afterwards incubated for 30 min with **(A, B)** MHC-I blocking antibody W6/32 at indicated doses or **(C)** with an IgG control antibody (20 µg/ml) or ICAM-1 blocking antibody at indicated doses. Then EwS cells were co-cultured with HLA-A*02:01/CHM1^319^-specific allorestricted TCR transgenic CD8^+^ T cells (CHM1^319^ CD8^+^ T cells) or unspecific CD8^+^ T cells at effector to target ratios of **(A, C)** 1:1 and **(B)** 1:4 for 24 h. Representative results from three independent experiments with three independent donors in **(A, B)** triplicates or **(C)** quadruplicates are displayed. Data are presented as mean ± SD. Two-way ANOVA with multiple comparison Šidák test was used to calculate p values. ns = not significant, * p ≤ 0.05, ** p ≤ 0.01, *** p ≤ 0.001, **** p ≤ 0.0001.

To mechanistically examine if TNF-induced upregulation of MHC-I on EwS cells is important for the enhanced T cell recognition and killing, the recognition of TNF-pretreated EwS cells was assessed in the presence of MHC-I blocking antibody (W6/32). The IFNγ release of CHM1^319^ CD8^+^ T cells was dose-dependently abrogated after blockade of MHC-I on A673 and TC32 using the anti-MHC-I antibody W6/32 (**Figures 7A, B**, top panels), confirming MHC-I specificity of CHM1^319^ CD8^+^ T cells. The pretreatment of A673 and TC32 with TNF significantly increased the IFNγ release from CHM1^319^ CD8^+^ T cells. This increased IFNγ release remained under partial MHC-I blockade on A673, but not TC32, thus indicating that TNF enhances T cell recognition of EwS cells by upregulating MHC-I. As recognition of TNF-pretreated TC32 did not remain increased under partial MHC-I blockade, although TNF dose-dependently upregulated MHC-I on TC32 (**Figure 3**), additional co-stimulatory surface molecules may contribute to the enhanced recognition of TNF-pretreated A673 by CHM1^319^ CD8^+^ T cells. In line with TNF-pretreatment of pediatric sarcoma cell lines not promoting anti-tumor activity of unspecific PBMCs (**Figure 5**), TNF-pretreatment of A673 and TC32 did not enhance IFNγ release of unspecific CD8^+^ T cells (**Figures 7A, B**, bottom panels), further indicating that TNF does not promote MHC-I independent cytotoxicity.

Since recognition of TNF-pretreated A673 remained enhanced under partial MHC-I blockade and TNF also strongly upregulated ICAM-1 on A673, we tested if membranous ICAM-1 contributes to the improved recognition by EwS antigen-specific transgenic T cells. Indeed, ICAM-1 blockade with 20µg/ml of the anti ICAM-1 antibody (clone P2A4) reduced the recognition of A673 pretreated with TNF by CHM1^319^ CD8^+^ T cells (**Figure 7C**), suggesting that ICAM-1 is involved in the enhanced recognition by antigen-specific CD8^+^ T cells. In line with the minor upregulation of ICAM-1 on TC32 by TNF pretreatment, ICAM-1 blockade did not affect recognition of TC32 regardless of whether the cells were pre-treated with TNF or not (**Figure 7C**). In addition to upregulation of immunogenic surface markers, altered antigen expression can also affect the recognition of EwS cell lines by CHM1^319^ CD8^+^ T cells. Therefore, we measured the mRNA expression of CHM1 in A673 and TC32 EwS cell lines after TNF pretreatment. As TNF significantly reduced mRNA expression of CHM1 in A673, but not in TC32 (**Supplementary Figure 13**), antigen expression and peptide loading on MHC-I may not be involved in the enhanced recognition and lysis of A673 cells after pretreatment with TNF.

Together, these results indicate that TNF sensitizes the EwS cell line A673 depending on the presence of MHC-I and upregulation of ICAM-1 for the recognition and lysis by CHM1^319^ CD8^+^ T cells without enhancing exhaustion.

### 3.3 A673 is the most responsive EwS cell line to pro-inflammatory cytokines

**Figure 8:**
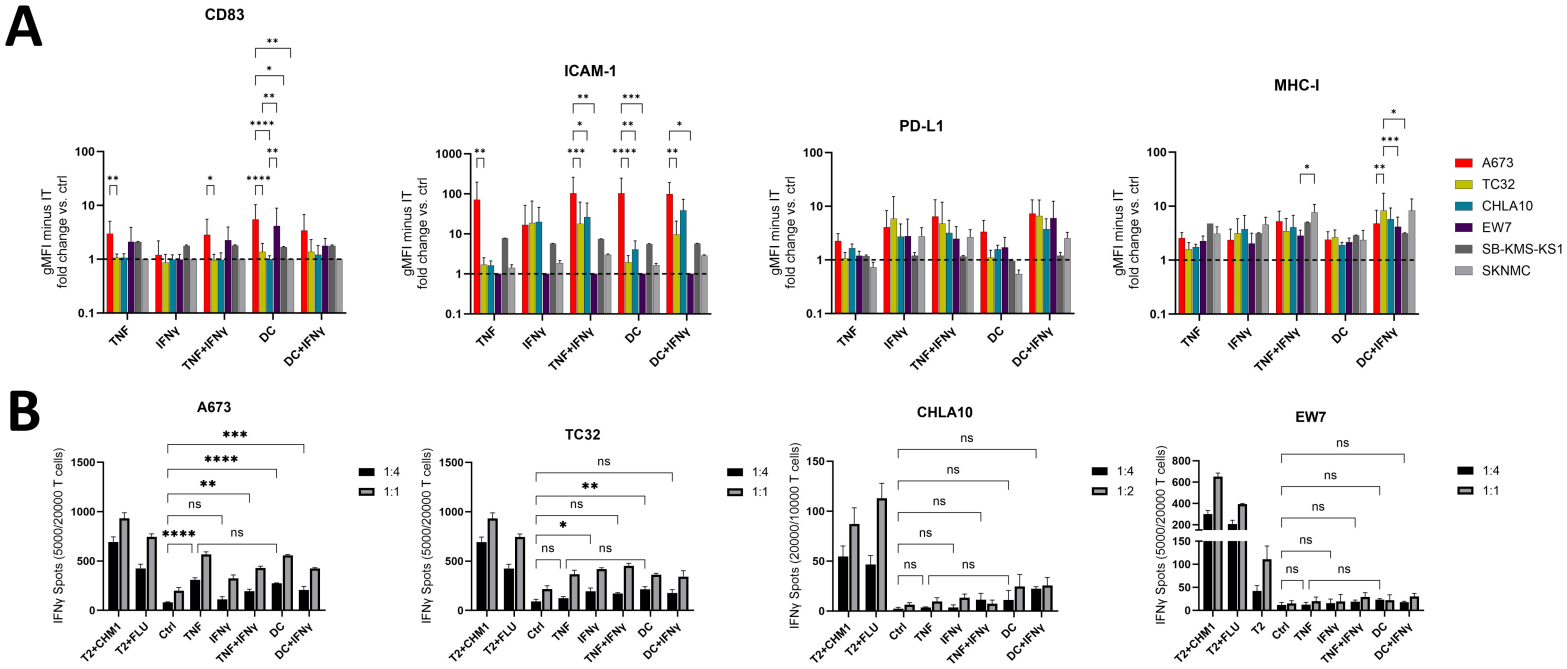
Pretreatment with TNF or monocyte maturation mediators sensitizes A673 and partly TC32 for the recognition by CHM1^319^ CD8^+^ T cells. **(A)** Flow cytometry of Ewing sarcoma (EwS) cell lines A673, TC32 and EW7 (all HLA-A2^+^) as well as CHLA10, SKNMC and SB-KMS-KS1 (all HLA-A2^-^) after pretreatment with TNF (10 ng/ml, 3 days) +/- IFNγ (100 U/ml, 1 day) or monocyte maturation mediators (DC cocktail: IL-4, GM-CSF for 5 days, followed by IL-1β, IL-6, TNF and PGE_2_ for 2 days) +/- IFNγ (100 U/ml, 1 day) or IFNγ (100 U/ml, 1 day) alone. Control (ctrl) cells were left untreated. Fold change over ctrl cells of the geometric mean fluorescence intensities (gMFI) minus the gMFI of the respective isotype ctrl is shown. Combined results from one (SB-KMS-KS1 and SKNMC), four (CHLA10), five (EW7), eight (A673) or nine (TC32) independent experiments with triplicates are shown. **(B)** ELISpot assay showing IFNγ spot count. EwS cells from **(A)** were co-cultured at indicated effector to target ratios with CHM1^319^-specific TCR-transgenic CD8^+^ T cells (CHM1^319^ CD8^+^ T cells) for 24 h. Representative results from two (CHLA10), four (A673, EW7) or five (TC32) independent experiments with triplicates are shown. Two-way ANOVA with multiple comparison Tukey´s test was used to calculate p values. ns = not significant, * p ≤ 0.05, ** p ≤ 0.01, *** p ≤ 0.001, **** p ≤ 0.001.

Since A673 cells showed superior sensitivity to lysis by EwS antigen-specific CD8^+^ T cells after TNF-pretreatment, we analyzed additional EwS cell lines, to identify those which can be sensitized using five different cytokine treatment combinations, including TNF. However, among a panel of six EwS cell lines tested by flow cytometry, A673 cells stood out as the strongest responders to treatment with TNF, TNF+IFNγ, DC and DC+IFNγ, especially in upregulating CD83, ICAM-1 and PD-L1 (**Figure 8A**). In contrast, MHC-I was upregulated at rather equal levels on the six EwS cell lines by the five treatment conditions. Prominent responses were observed in combinational treatments of IFNγ with TNF or the DC cocktail, upregulating ICAM-1, MHC-I and CD83 on three (A673, TC32 and CHLA10) and MHC-I on all six EwS cell lines (**Figure 8A**). However, these treatments also resulted in a strong upregulation of PD-L1.

Further analysis of HLA-A2^+^ (A673, TC32 and EW7) and HLA-A2^-^ (CHLA10, SB-KMS-KS1 and SKNMC; **Supplementary Figure 11**) EwS cell lines by IFNγ ELISpots showed that pretreatment with TNF reliably enhanced the recognition of A673 by CHM1^319^ CD8^+^ T cells, while recognition of TC32 was improved at E:T ratios of 1:1. In contrast, EW7 and the HLA-A2^-^ cell lines CHLA10, SB-KMS-KS1 and SKNMC failed to respond (**Figure 8B**, **Supplementary Figure 14**). TNF efficiency was comparable to that of the DC cytokine cocktail, which enhanced sensitivity of A673 and partly TC32 (3/5 independent experiments; **Figure 8B**).

**Figure 9:**
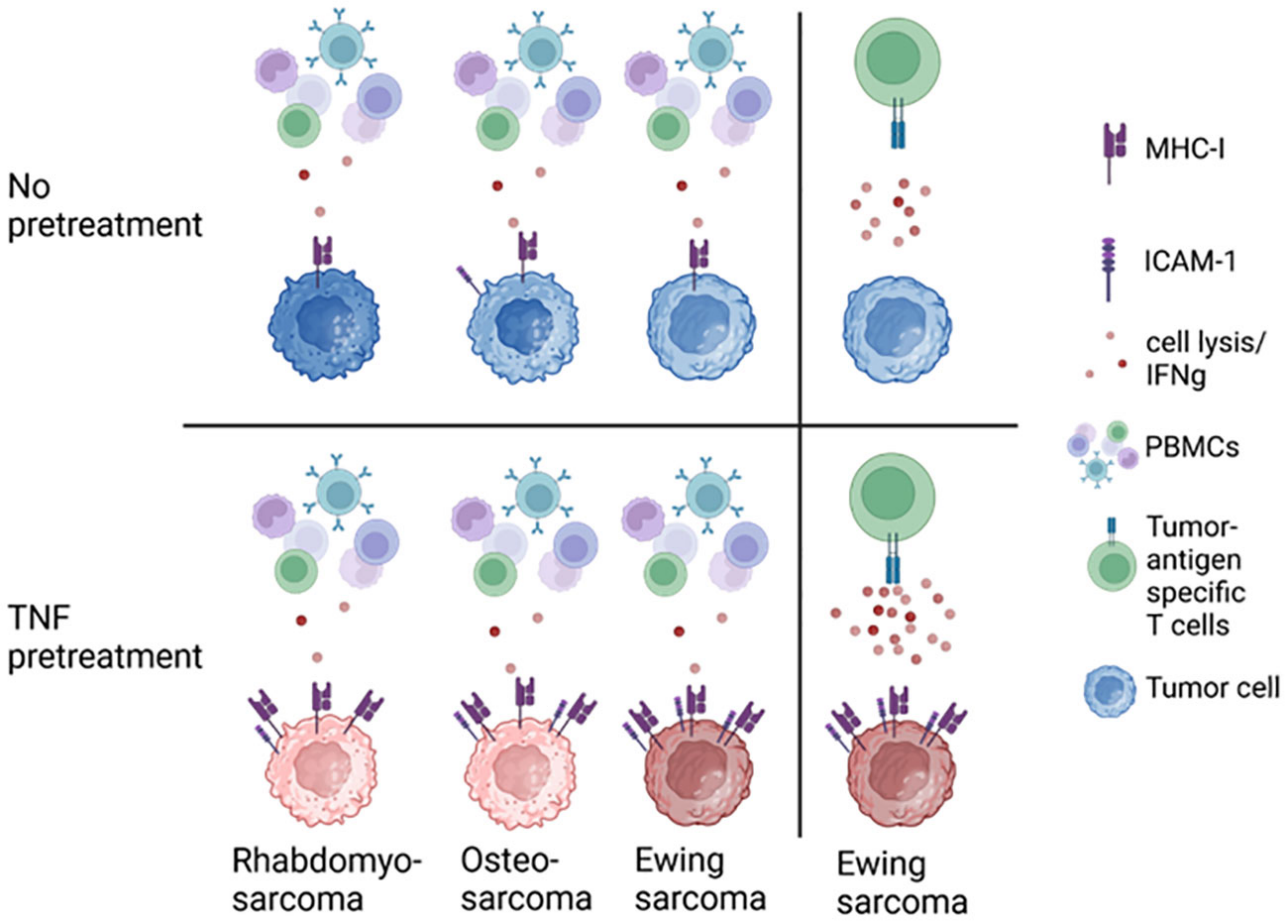
TNF pretreatment upregulates MHC-I and ICAM-1 expression on pediatric sarcoma cell lines, sensitizing Ewing sarcoma to tumor antigen-specific CD8^+^ T cells.

In summary, pretreatment of EwS cell lines with TNF, IFNγ, DC maturation mediators or their combinations with IFNγ induce distinct patterns of immunogenic and immunosuppressive cell surface markers. TNF appears to be the strongest and most advantageous as monotreatment, based upon its ability to firmly induce the immunostimulatory surface markers MHC-I, ICAM-1 and CD83. Among three HLA-positive EwS cell lines tested, A673 cells exhibited a defined response, including upregulation of immunostimulatory markers and enhanced sensitivity to antigen-specific T cells, while TC32 cells showed partial response and EW7 were non-responders. The responsiveness of A673 depends on MHC-I and partly ICAM-1, providing a rational to induce MHC-I and ICAM-1 expression to improve the efficacy of adoptive cellular therapies in pediatric sarcomas (**Figure 9**).

## 4 Discussion

Most pediatric sarcomas are non-T cell-inflamed tumors, with 5-year survival rates less than 20-30% in patients with metastatic disease. So far, immunotherapies have not yielded strong clinical responses in pediatric sarcomas, despite their success in T cell-infiltrated immunogenic tumors (9). In this work, we sought to test strategies to enhance immunogenicity in general and specifically the sensitivity of pediatric sarcomas for adoptive T cell transfer by inducing immunogenic cell surface markers. We used a panel of EwS, RMS and OS pediatric sarcoma cell lines to test six cytokines, used for monocyte differentiation and DC maturation (IL-4, GM-CSF, IL-1β, IL-6, TNF and PGE_2_), for their ability to induce cancer cell immunogenicity and sensitivity to tumor antigen-specific T cells.

Applying flow cytometry, immunoblotting, tumor killing and ELISpot assays, we identified TNF and IL-1β to be the strongest inducers of MHC-I, and partly ICAM-1 and CD83 on the majority of pediatric sarcoma cell lines tested. Especially TNF was superior for upregulating immunostimulatory cell surface molecules and sensitizing EwS cells to tumor antigen-specific T cells. In contrast to TNF and IL-1β, the other tested monocyte maturation mediators, including IL-4, GM-CSF, IL-6 and PGE_2_, weakly affected expression of immunogenic surface markers on pediatric sarcoma cells. This is in accordance with clinical trials, which failed to show clinical benefit in response to administration of GM-CSF in EwS and OS (27–29). Despite expression of IL-6R and IL-6ST on EwS cell lines (30), IL-6 did not modulate expression of immunogenic markers on pediatric sarcoma cell lines. This suggests that IL-6R-pSTAT3 signaling is not involved in the upregulation of immunogenic markers in pediatric sarcomas but prevents apoptosis and promotes migration in pediatric sarcomas (30–32).

TNF enhanced the recognition of EwS cell line A673 and to a lesser extent TC32 by CHM1^319^ CD8^+^ T cells, but not to HLA-mismatched healthy donor-derived PBMCs, indicating that MHC-I peptide specific TCR repertoires are required for the effect of TNF. Indeed, MHC-I blockade by the MHC-I blocking antibody W6/32 demonstrated that the enhanced recognition of antigen-specific CD8^+^ T cells against A673 and TC32 depends on TNF-induced MHC-I upregulation. However, even in the specific TCR context, TNF only improved the lysis of one out of three HLA-A2-positive EwS cells against CHM1^319^ CD8^+^ T cells despite MHC-I upregulation. The blockade of ICAM-1 showed that TNF-induced membranous ICAM-1 contributes to the enhanced recognition of A673 by CHM1^319^ CD8^+^ T cells. This is in line with the membranous ICAM-1 promoting T cell binding and immune synapse formation (33, 34), as well as enhanced cytotoxicity of antigen-specific T cells (35). Yet, further experiments, including ICAM-1 knockout experiments, are required for a final conclusion regarding the role of ICAM-1 in the recognition of TNF-pretreated EwS cells by CHM1^319^ CD8^+^ T cells. As TNF downregulated the expression of the target antigen CHM1, altered antigen expression, processing and loading on MHC-I may have not contributed to the TNF-induced enhanced lysis of A673 by CHM1^319^ CD8^+^ T cells. Other potential mechanisms may include differential regulation of TNF signaling, receptors of the TNF superfamily (36), or other regulatory genes or pathways, variations in EWS-FLI1 protein levels (25) or metabolic states, each of which requires further investigation. The most interesting possibility would be if A673 represents a subset of EwS tumors, which are more susceptible for adoptive T cell transfer and other types of immunotherapy.

Although the efficacy of TNF and IFNγ in upregulating MHC-I on OS and EwS was previously reported (37, 38), our study demonstrates that TNF may be advantageous for T cell-based immunotherapies. As a potential mechanism, TNF signals through TNFR1 to activate the NFκB pathway, which can induce MHC-I and ICAM-1 expression (39, 40). In turn, upregulation of MHC-I improves recognition of tumor cells by cytotoxic T cells (40, 41). In the tumor microenvironment, the interplay of TNF and IFNγ can be a prerequisite for controlling tumor development (42). Indeed, in OS and EwS patients tumor infiltrating CD14^+^CD16^+^ myeloid cells show a TNF signaling signature (43). However, despite their presence in the TME, the T cell tumor infiltration is low (7, 8) and response to immune checkpoint blockade is poor, indicating that TNF and IFNγ present in the TME are insufficient to stimulate an effective anti-tumor immunity.

The systemic administration of TNF in humans showed systemic toxicity and low anti-tumor response (44–47), while localized administration in the form of isolated limb perfusion in combination with chemotherapeutics exerts clinical responses and improves limb salvage (48). Here, TNF sensitized the EwS cell line A673 to antigen-specific CD8^+^ T cells. Hence, a translational approach could be to stimulate the local release of TNF from tumor cells or the tumor microenvironment (49, 50) shortly before adoptive T cell transfer, enhancing tumor cell immunogenicity and T cell therapy efficacy. TNF exerts pleiotropic effects on T cells depending on their cell state, including TCR-dependent activation, cytokine production, proliferation, apoptosis or promoting immunosuppression (51). Here, we did not assess the direct effect of TNF on antigen-specific CD8^+^ T cells. As the next step, the effects of TNF-inducing regimen on the T cell infiltration, phenotype, activation, anti-tumor cytotoxicity and exhaustion, as well as the tumor microenvironment should be evaluated in suitable *in vivo* models.

The findings of this study highlight that established pediatric sarcomas differ in their immunological properties even within a tumor entity, emphasizing the importance of selecting a panel of cell lines in the functional testing of immunotherapies, including T cell therapies. This is in line with EwS cell lines showing a stable but variable degree of genome, transcriptome and phenotype (52). In summary, this study identifies TNF as a potential treatment opportunity for enhancing immunogenicity of pediatric sarcomas without promoting tumor growth. Among all pediatric sarcoma cell lines tested, A673 EwS cells were the most responsive and the TNF-induced upregulation of MHC-I and ICAM-1 sensitized A673 for the lysis by CHM1^319^ CD8^+^ T cells. Further studies should evaluate the role of TNF and TNF-inducing regimens *in vivo* as a neoadjuvant sensitizing upfront adoptive T cell transfer in pediatric sarcomas.

## Data Availability

The raw data supporting the conclusions of this article will be made available by the authors, without undue reservation.
