# Supplementary material for: Cytokine screening identifies TNF to potentially enhance immunogenicity of pediatric sarcomas
Source: Front Immunol. 2024 Dec 11;15:1347404. doi: 10.3389/fimmu.2024.1347404 (PMC11668575; doi:10.3389/fimmu.2024.1347404)
Supplement: Supplementary file 1 [file DataSheet1.docx]

Supplementary Material

Cytokine screening identifies TNF to potentially enhance immunogenicity of pediatric sarcomas

Hendrik Gassmann^1,2,*^, Melanie Thiede^1^, Jennifer Weiß^1^, Emilie Biele^1^, Luisa Flohé, Helena Lachermaier^1^, Carolin Prexler, Valentina Evdokimova^3^, Laszlo Radvanyi^3^, Irfan Akhtar^1^, Mina N. F. Morcos^1^, Franziska Auer^1^, Sebastian J. Schober^1^, Julia Hauer^1,2^, Uwe Thiel^1^, Kristina von Heyking^1,*^

*** Correspondence:** hendrik.gassmann@tum.de, kristina.heyking@tum.de

# Supplementary Figures and Tables

For more information on Supplementary Material and for details on the different file types accepted, please see [here](https://www.frontiersin.org/guidelines/author-guidelines#supplementary-material).

## Supplementary Figures


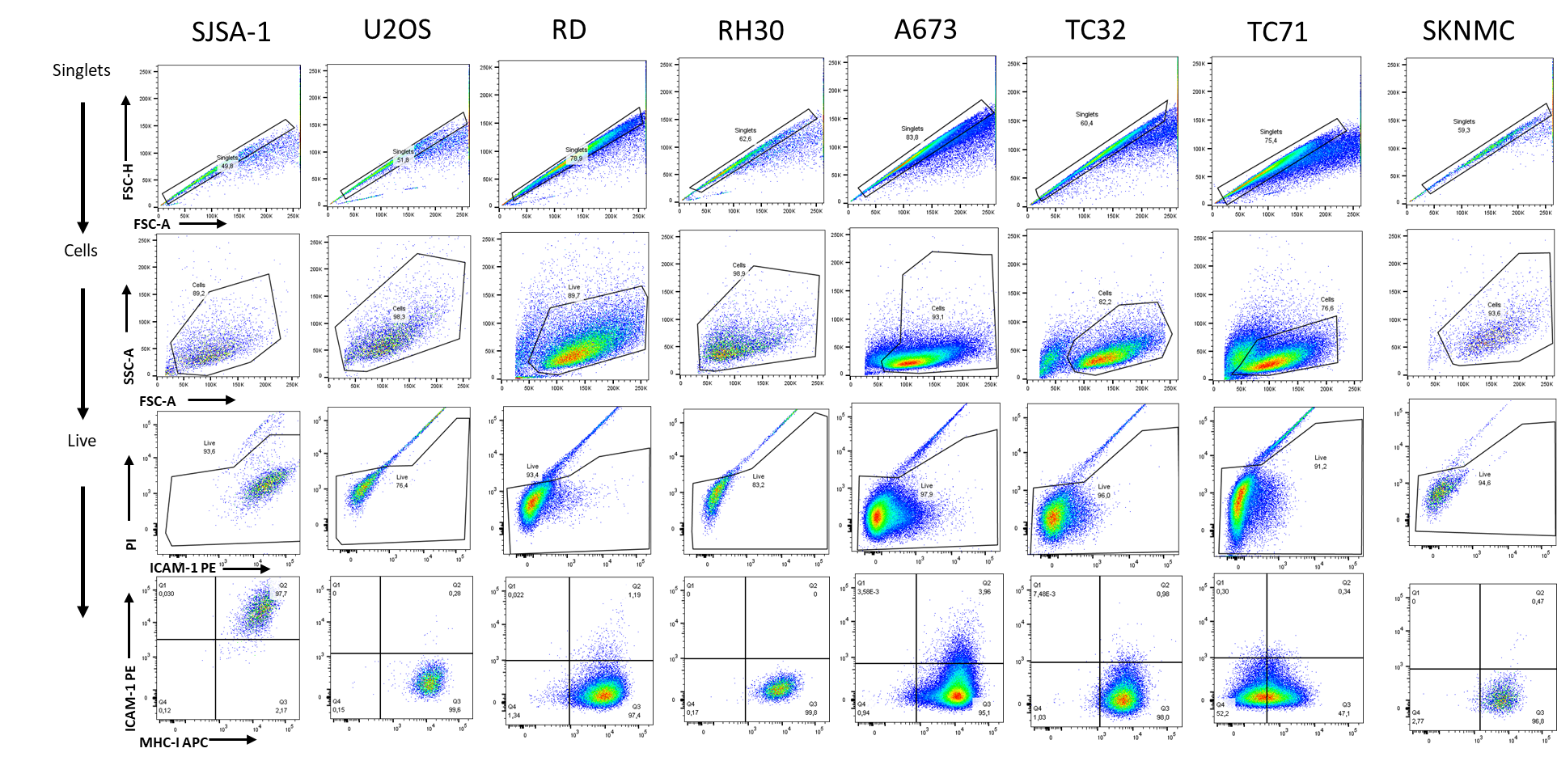


**Supplementary Figure 1.** **Gating strategy for flow cytometry of pediatric sarcoma cell lines**. Gating strategy for analyzing expression of surface markers of indicated pediatric sarcoma cell lines by flow cytometry. Gating was performed on Singlets (FSC-H vs. FSC-A), Cells (SSC-A vs. FSC-A) and Live cells (PI vs. PE). Isotype controls were used to assess specific binding, correct for background signals and normalization.

**
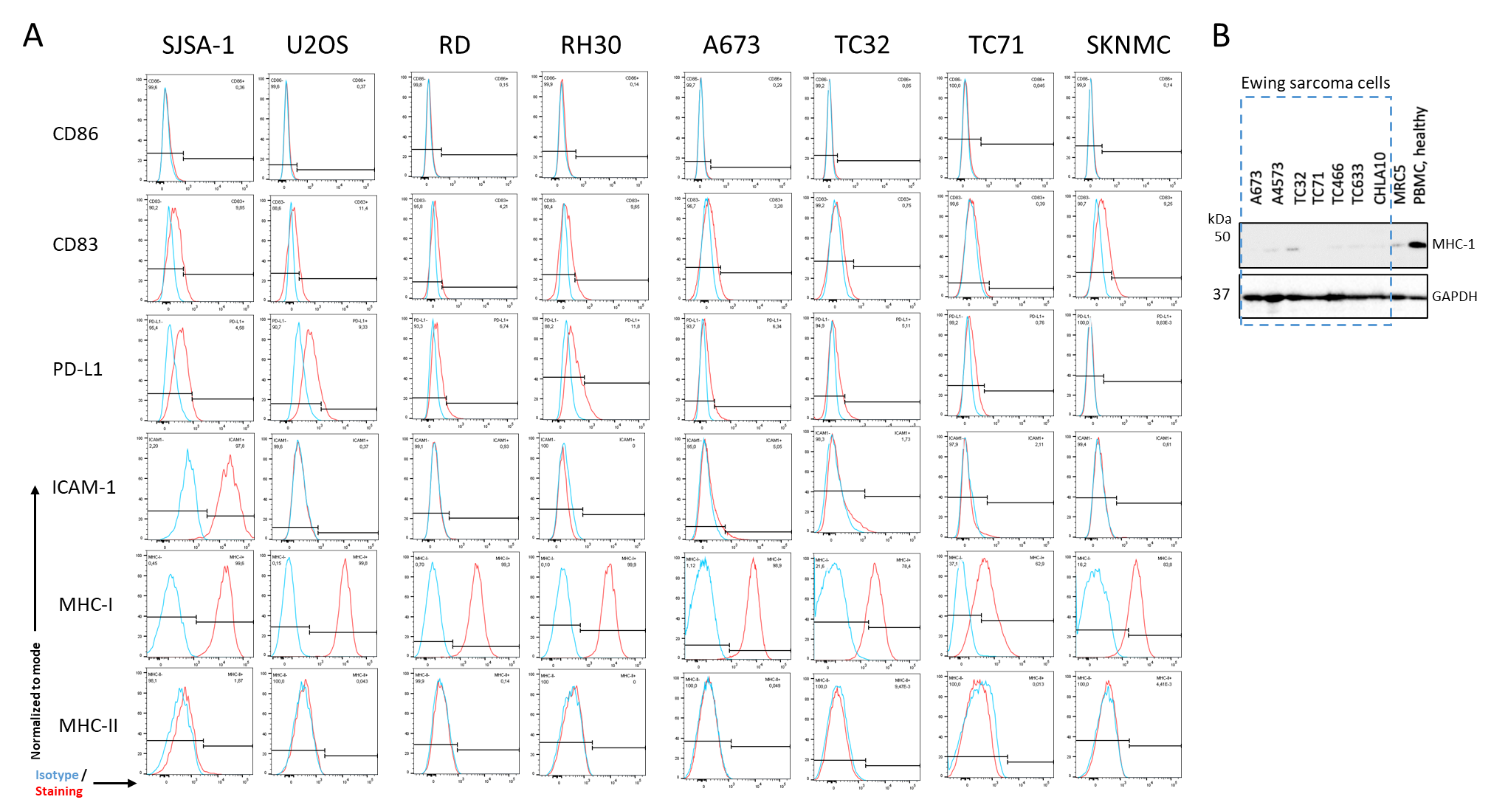
**

**Supplementary Figure 2.** **Pediatric sarcomas slightly express MHC-I and lack immunogenic surface markers.** (**A**) Flow cytometry of osteosarcoma (SJSA-1, U2OS), rhabdomyosarcoma (RD, RH30) and Ewing sarcoma (A673, TC32, TC71 and SKNMC) cell lines. Histograms normalized to mode displaying the expression of respective immunogenic surface markers (red) and corresponding isotype (IT, blue). (**B**) Western blot for MHC-I expression in indicated Ewing sarcoma cell lines, MRC5 fibroblasts and healthy donor-derived PBMCs. GAPDH was used as housekeeping protein. Representative results from one (B) or at least three (A) independent experiments with one (B) or three (A) replicates each are shown.


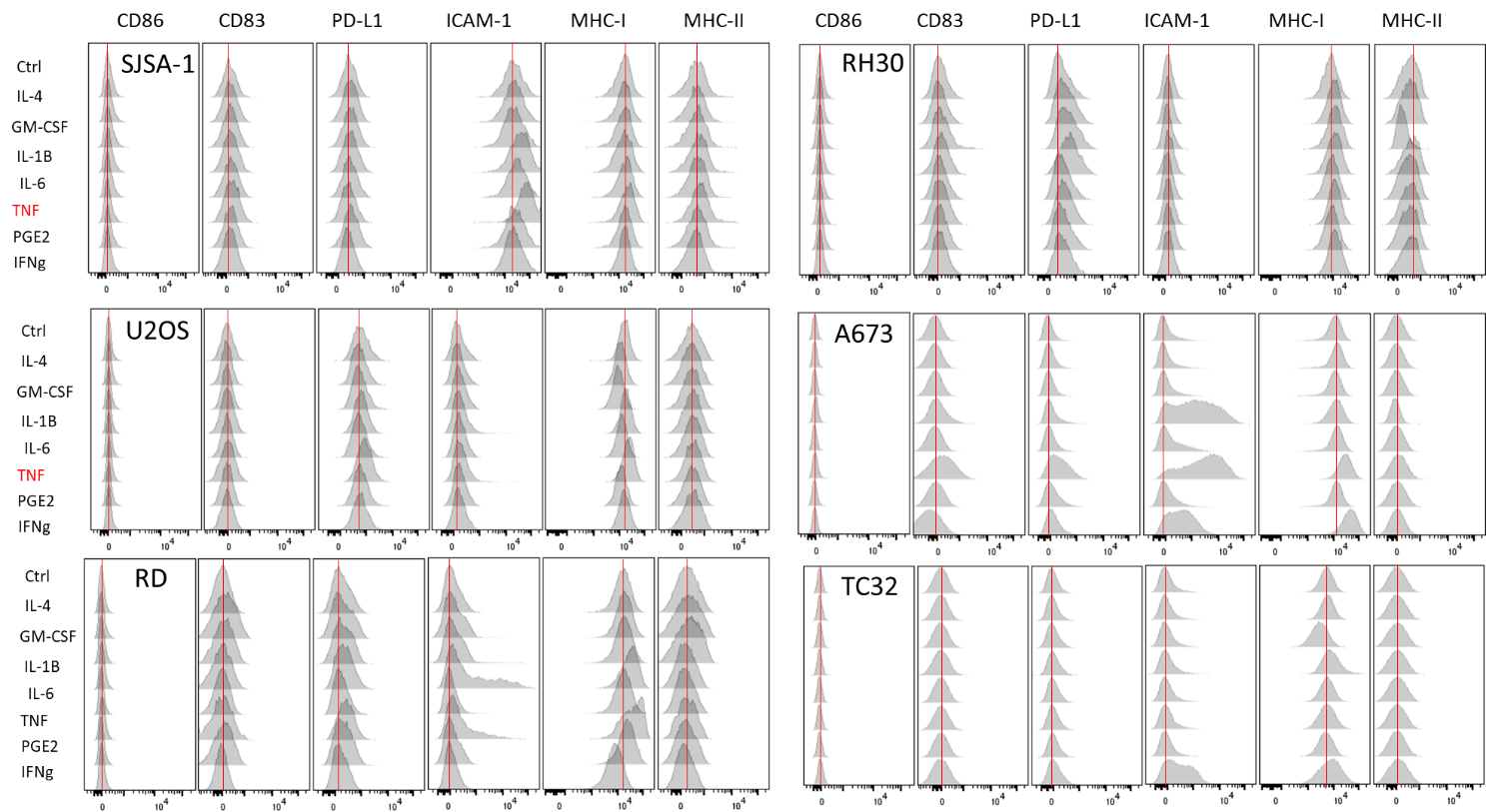


**Supplementary Figure 3.** **TNF and IL-1β are the major cytokines upregulating immunogenic markers on pediatric sarcoma cells.** Flow cytometry of selected pediatric sarcoma cell lines. Shown are histograms of immunogenic surface markers (gray) after 96 h treatment with indicated cytokines or left untreated as control (ctrl). Representative results from two independent experiments with three replicates each are shown.


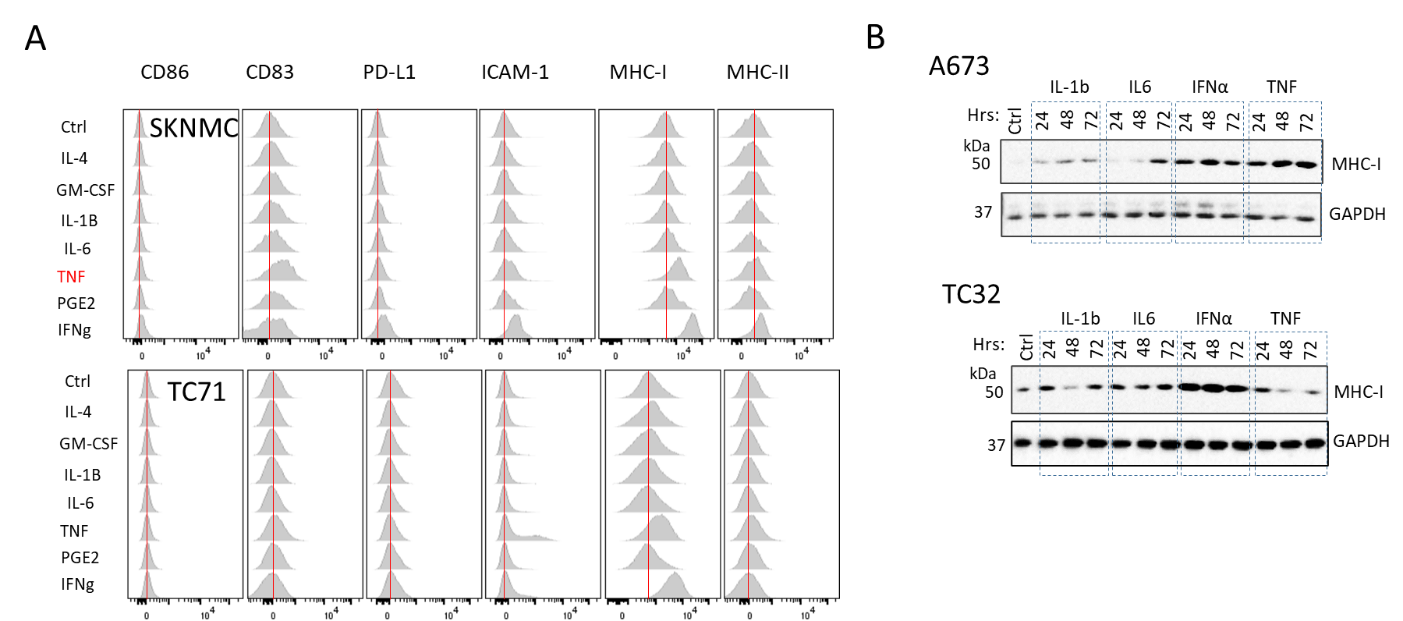


**Supplementary Figure 4.** **TNF and IL-1β are the major cytokines upregulating immunogenic markers on pediatric sarcoma cells. (A)** Flow cytometry of different Ewing sarcoma (EwS) cell lines. Shown are histograms of immunogenic surface markers (gray) after 96 h treatment with indicated cytokines or left untreated as control (ctrl). **(B)** Western blot for MHC-I in EwS cell lines A673 (top) and TC32 (bottom) after indicated duration of treatment with different cytokines. IFNα was used as positive control and GAPDH was used as housekeeping protein. Representative results from one (B) or three (A) independent experiments with one (B) or three (A) replicates each are shown.

**
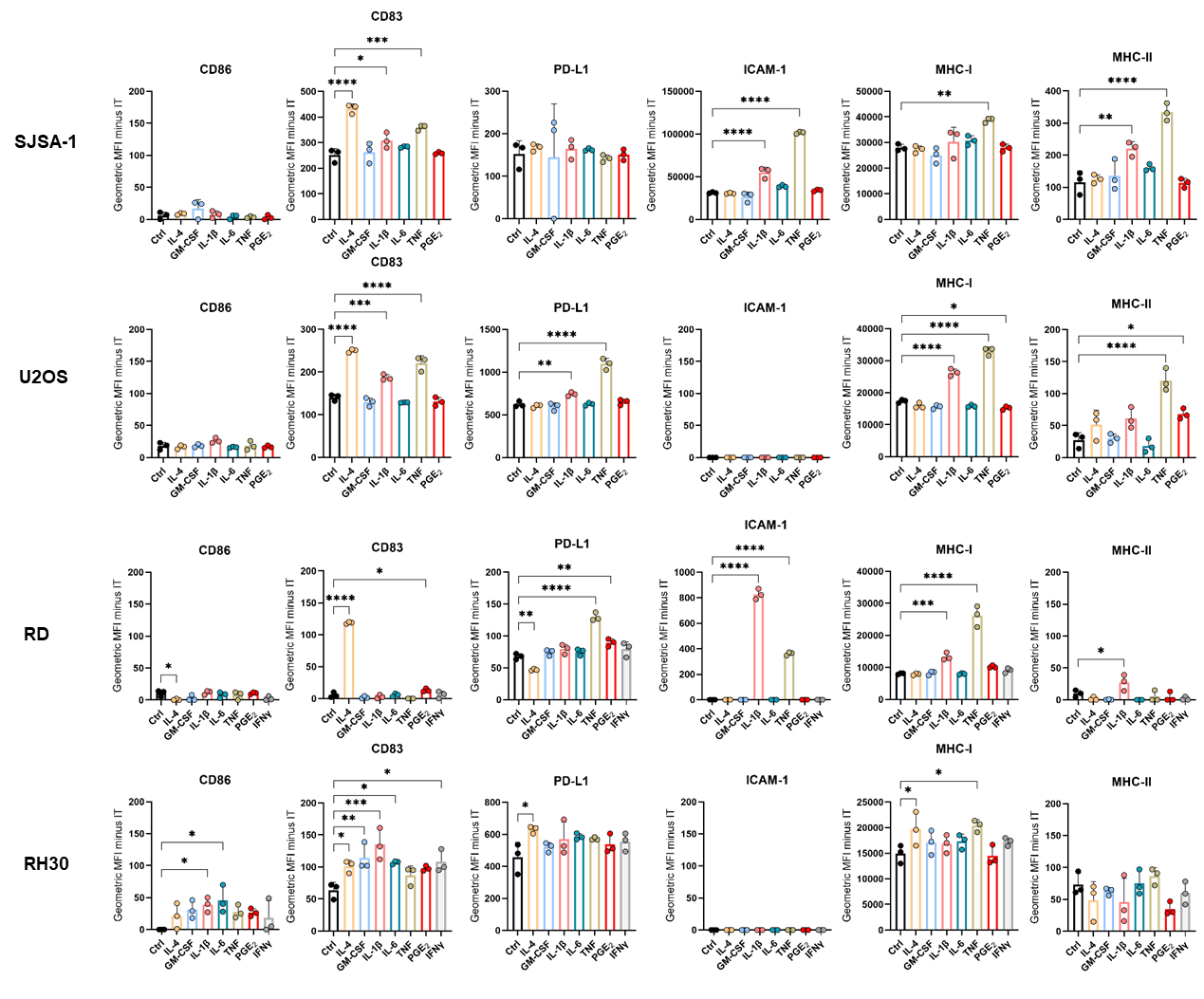
**

**Supplementary Figure 5.** **TNF and IL-1β are the major cytokines upregulating immunogenic markers on pediatric sarcoma cells.** Flow cytometry of four pediatric sarcoma cell lines after 96 h treatment with indicated cytokines or left untreated as control (ctrl). Shown is the expression of immunogenic surface markers as geometric mean fluorescence intensities (gMFI) of the specific staining minus gMFI of the respective isotype ctrl. Representative results from two independent experiments with three replicates each are shown. Data are presented as mean ± SD. One-way ANOVA with multiple comparison Dunnett´s test was used to calculate p values (E). * p ≤ 0.05, ** p ≤ 0.01, *** p ≤ 0.001, **** p ≤ 0.0001.

**
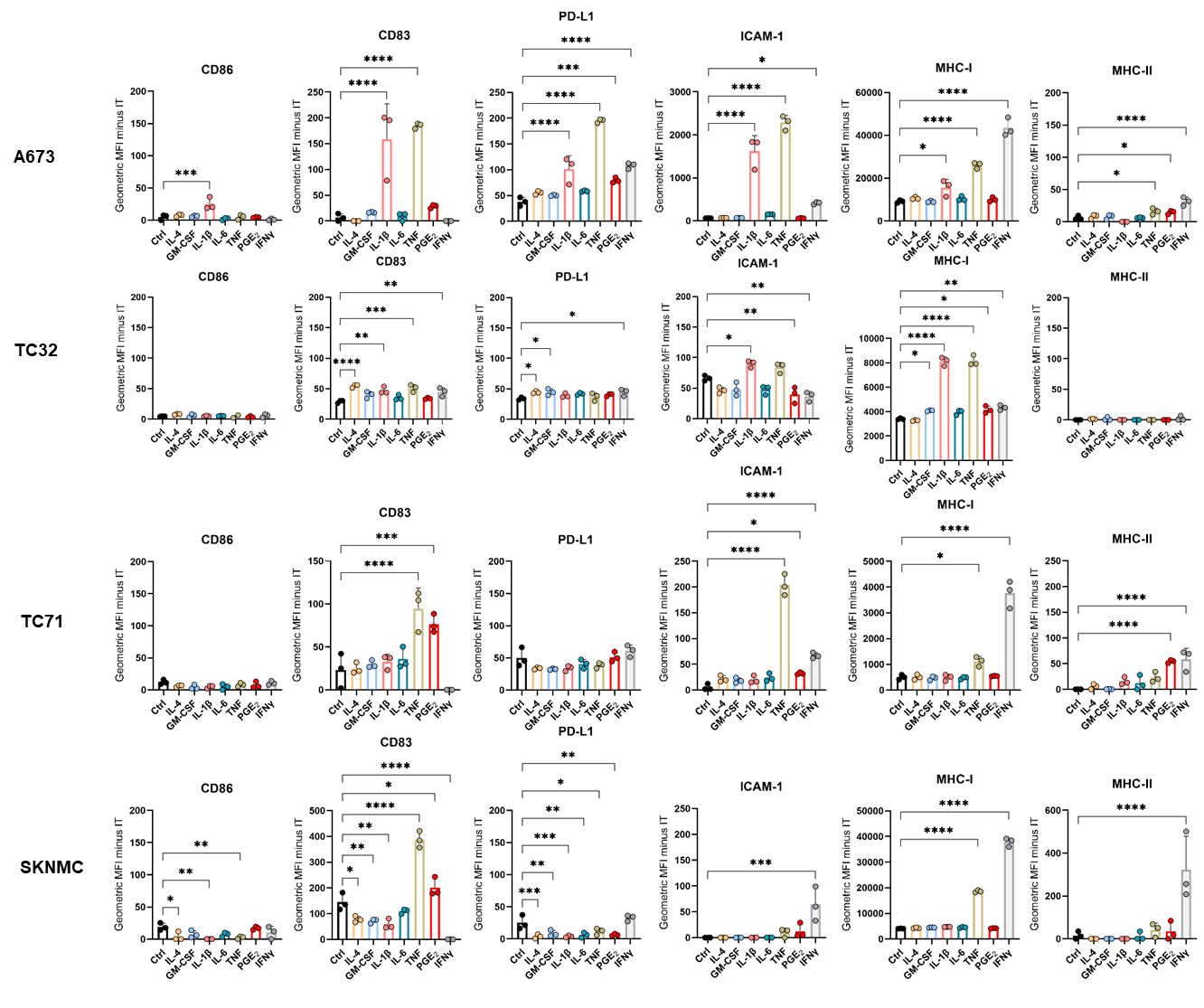
**

**Supplementary Figure 6.** **TNF and IL-1β are the major cytokines upregulating immunogenic markers on Ewing sarcoma cells.** Flow cytometry of four pediatric sarcoma cell lines after 96 h treatment with indicated cytokines or left untreated as control (ctrl). Shown is the expression of immunogenic surface markers as geometric mean fluorescence intensities (gMFI) of the specific staining minus gMFI of the respective isotype ctrl. Representative results from two independent experiments with three replicates each are shown. Data are presented as mean ± SD. One-way ANOVA with multiple comparison Dunnett´s test was used to calculate p values (E). * p ≤ 0.05, ** p ≤ 0.01, *** p ≤ 0.001, **** p ≤ 0.0001.

**
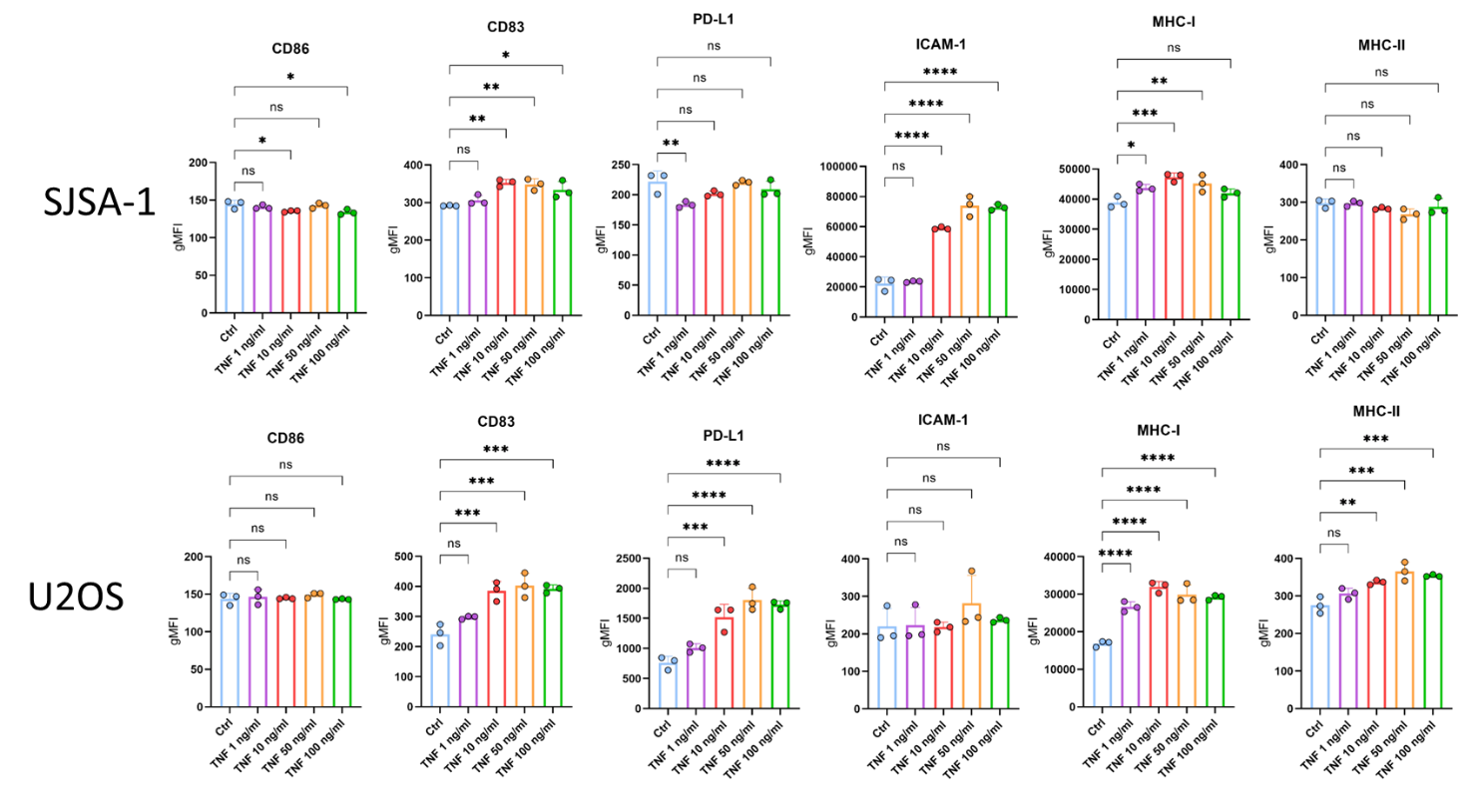
**

**Supplementary Figure 7.** **TNF dose-dependently upregulates immunogenic markers on osteosarcoma cell lines.** Flow cytometry of two osteosarcoma cell lines after 96 h treatment with indicated doses of TNF or left untreated as control (ctrl). Shown is the expression of immunogenic surface markers as geometric mean fluorescence intensities (gMFI). Representative results from three independent experiments with three replicates are shown. Data are presented as mean ± SD. One-way ANOVA with multiple comparison Dunnett´s test was used to calculate p values. ns = not significant, * p ≤ 0.05, ** p ≤ 0.01, *** p ≤ 0.001, **** p ≤ 0.0001.


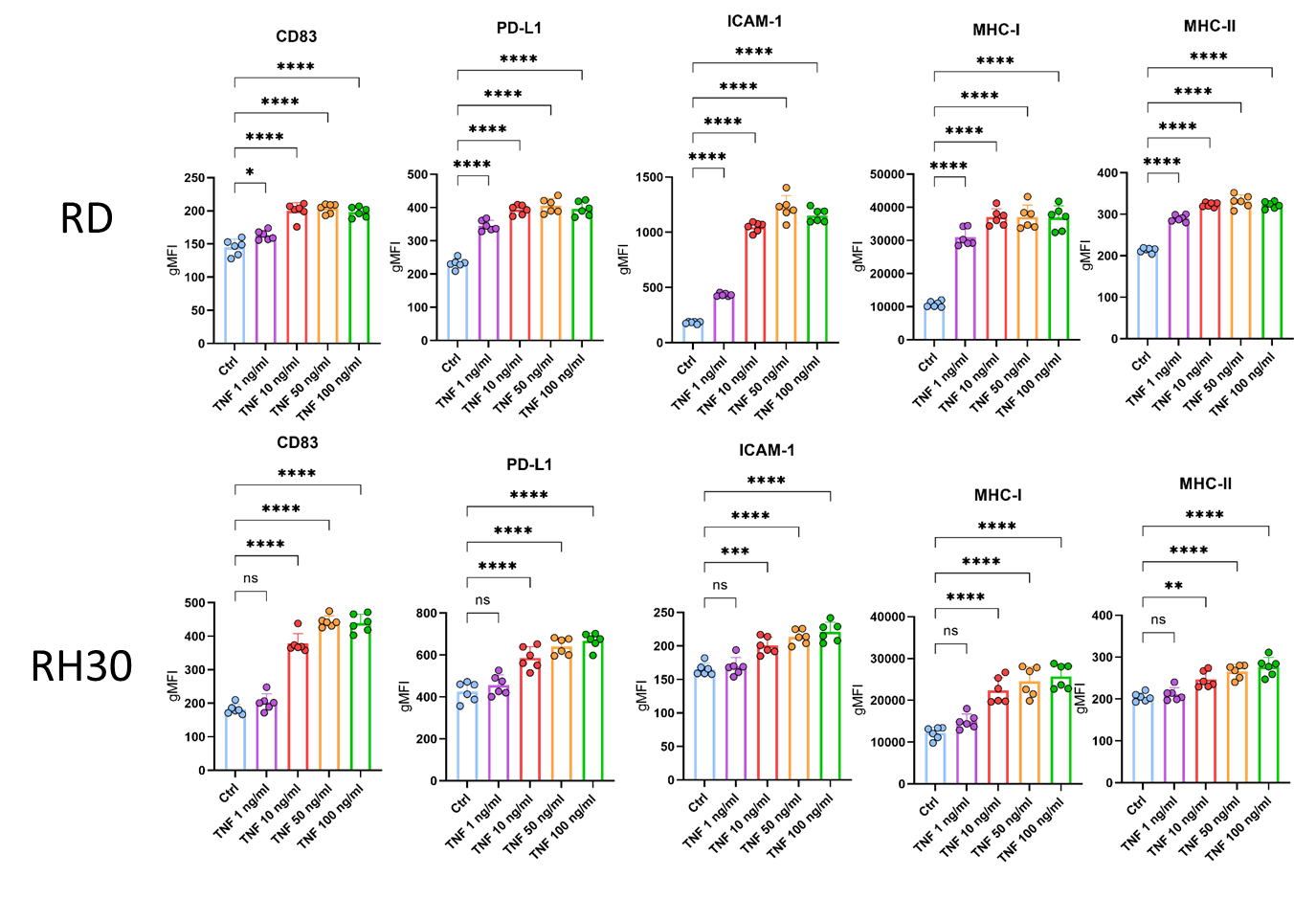


**Supplementary Figure 8.** **TNF dose-dependently upregulates immunogenic markers on rhabdomyosarcoma cell lines.** Flow cytometry of two rhabdomyosarcoma cell lines after 96 h treatment with indicated doses of TNF or left untreated as control (ctrl). Shown is the expression of immunogenic surface markers as geometric mean fluorescence intensities (gMFI). Representative results from three independent experiments with six replicates are shown. Data are presented as mean ± SD. One-way ANOVA with multiple comparison Dunnett´s test was used to calculate p values. ns = not significant, * p ≤ 0.05, ** p ≤ 0.01, *** p ≤ 0.001, **** p ≤ 0.0001.

**
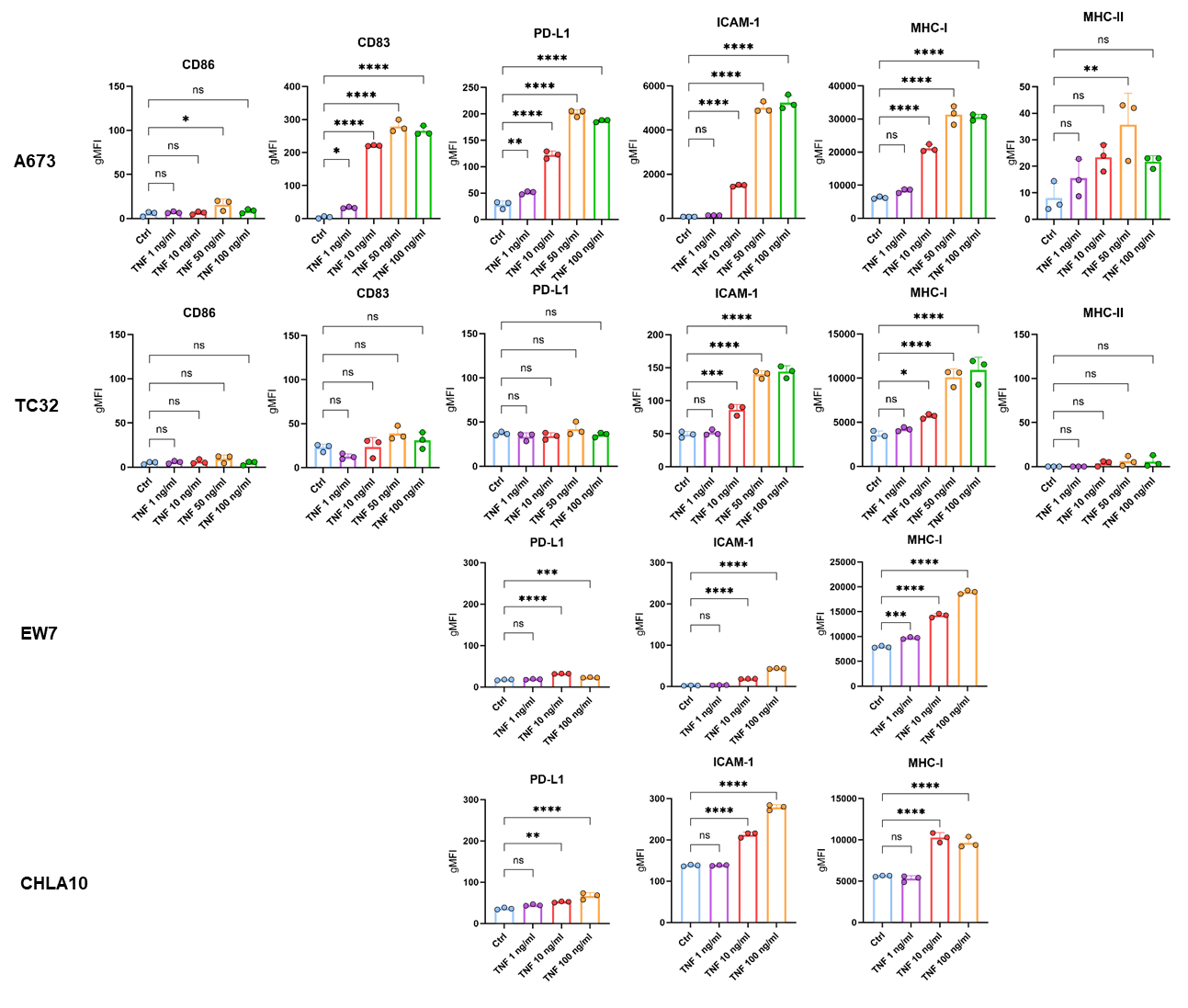
**

**Supplementary Figure 9.** **TNF dose-dependently upregulates immunogenic markers on Ewing sarcoma cell lines.** Flow cytometry of four Ewing sarcoma (EwS) cell lines after 96 h treatment with indicated doses of TNF or left untreated as control (ctrl). Shown is the expression of immunogenic surface markers as geometric mean fluorescence intensities (gMFI). Representative results from one (EW7 and CHLA10) or three (A673 and TC32) independent experiments with three replicates are shown. Data are presented as mean ± SD. One-way ANOVA with multiple comparison Dunnett´s test was used to calculate p values. ns = not significant, * p ≤ 0.05, ** p ≤ 0.01, *** p ≤ 0.001, **** p ≤ 0.0001.


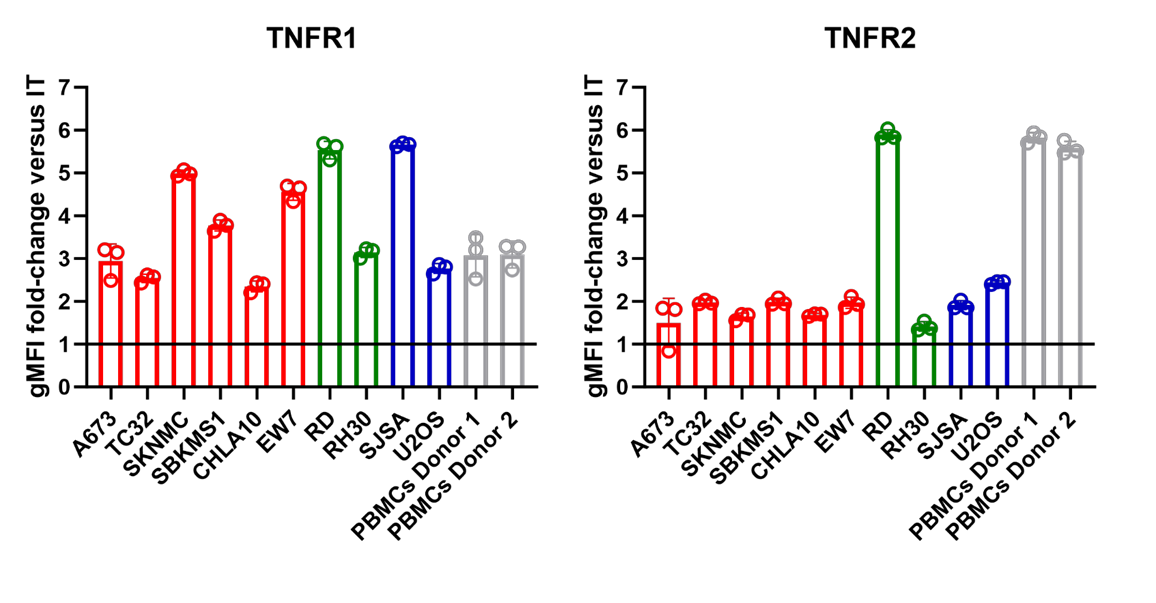


**Supplementary Figure 10. Pediatric sarcoma cell lines show modest to strong TNFR1 expression, while TNFR2 is only weakly expressed.** Flow cytometry showing surface expression of TNFR1 (left panel) and TNFR2 (right panel) on pediatric sarcoma cell lines compared to healthy donor PBMCs. Fold-change of the geometric mean fluorescence intensity (gMFI) versus the isotype (IT) control is displayed. A fold-change of one represents an absent expression. Results of three replicates are shown.


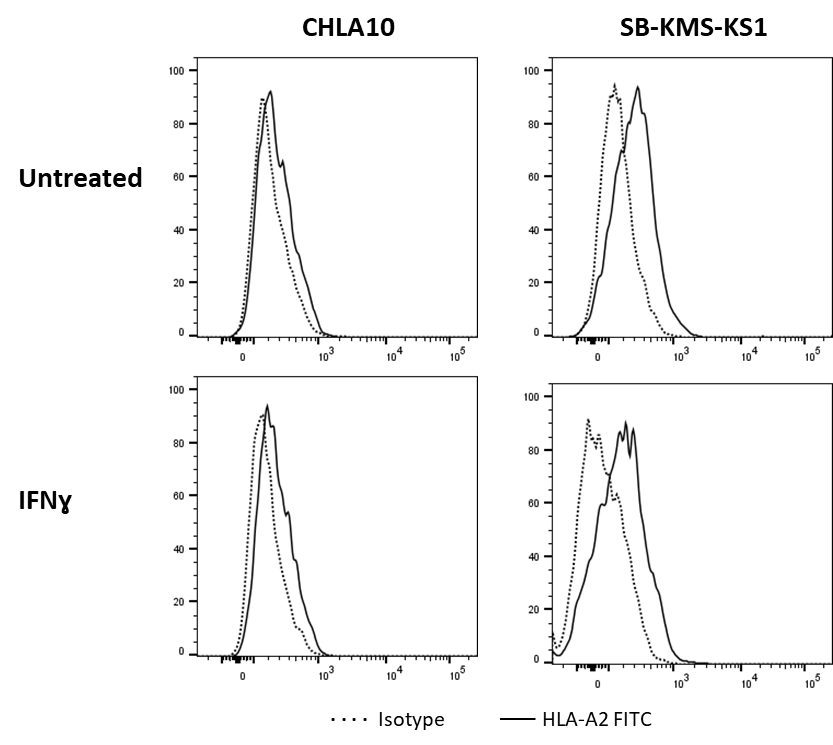


**Supplementary Figure 11.** **Ewing sarcoma cell lines CHLA10 and SB-KMS-KS1 are HLA-A2 negative.** Ewing sarcoma (EwS) cell lines CHLA10 (left panels) and SB-KMS-KS1 (right panels) were pretreated with IFNɣ (100 U/ml) (bottom panel) or left untreated (top panel). After 24 h cells were stained with anti-HLA-A2-FITC (solid line) or isotype control (dashed line) and analyzed by flow cytometry. Shown is the expression normalized to mode. Result from one independent experiment is presented.


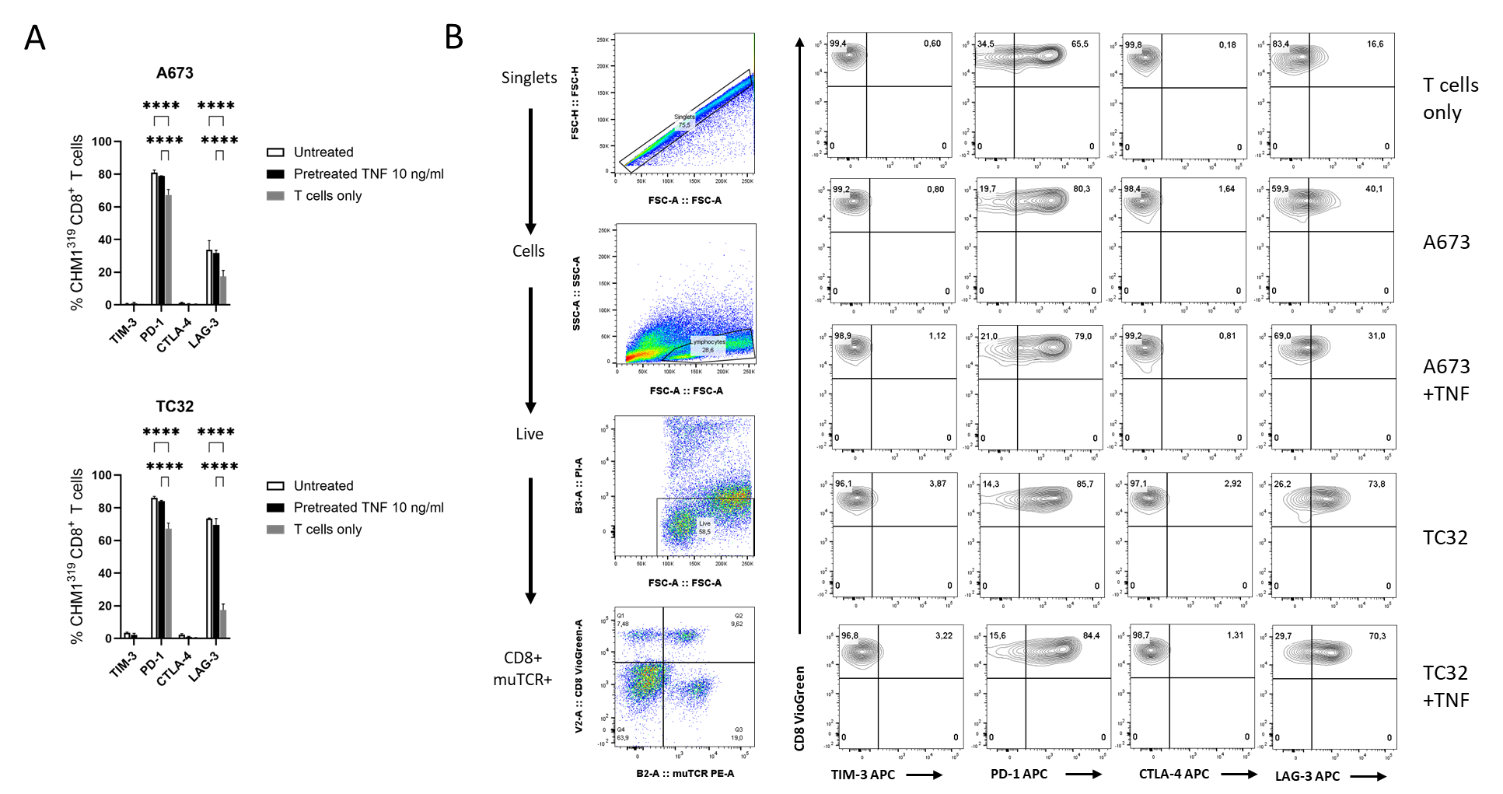


**Supplementary Figure 12. TNF-pretreatment of A673 and TC32 does not enhance exhaustion in antigen-specific CD8^+^ T cells.** (**A, B**) Flow cytometry of exhaustion markers TIM-3, PD-1, CTLA-4 and LAG-3 on CHM1^319^-specific TCR-transgenic CD8^+^ T cells (CHM1^319^ CD8^+^ T cells) after co-culture at an effector to target ratio of 1:2 with TNF-pretreated (10ng/ml for 72 h) A673 and TC32 for 96 h. Representative donor of six independent donors is shown. Data are presented as mean ± SD. Two-way ANOVA with Tukey´s multiple comparisons test was used to calculate p values. * p ≤ 0.05, ** p ≤ 0.01, *** p ≤ 0.001, **** p ≤ 0.0001.

**
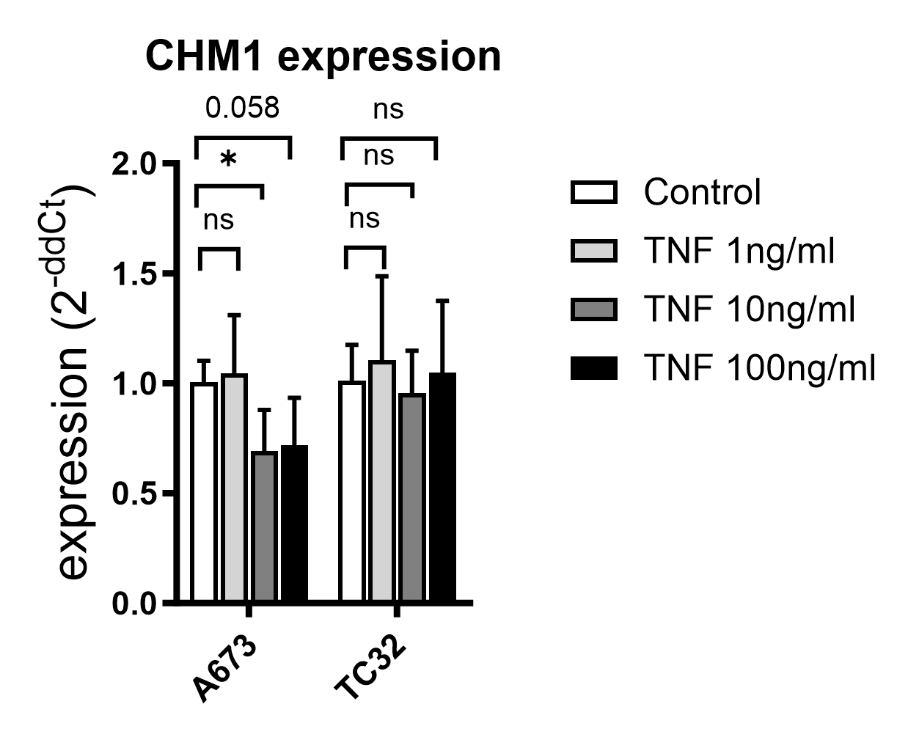
**

**Supplementary Figure 13. TNF downregulates CHM1 expression in A673 but not TC32.** sqRT-PCR showing CHM1 expression in A673 and TC32 after treatment with different concentrations of TNF (1-100 ng/ml) for 72 h. Combined results from three independent experiments with triplicates are shown. One-way ANOVA with multiple comparison Dunnett´s test was used to calculate p values (B). ns = not significant, * p ≤ 0.05, ** p ≤ 0.01, *** p ≤ 0.001, **** p ≤ 0.0001.


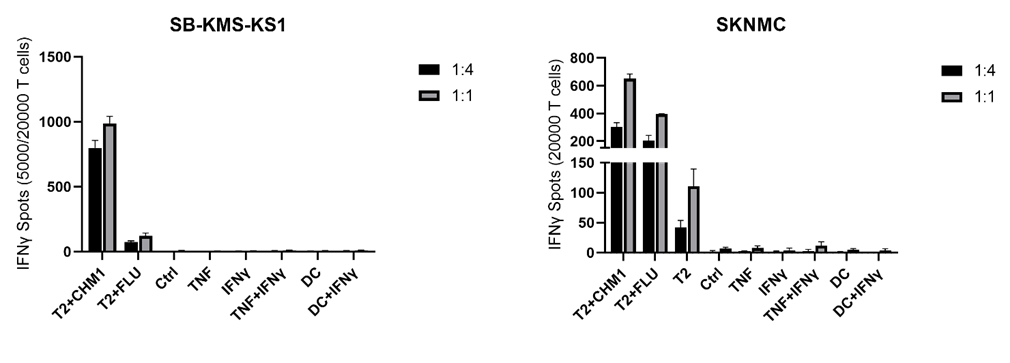
**Supplementary Figure 14.** **Pretreatment with TNF or monocyte maturation mediators does not sensitize HLA-A2 negative EwS cell lines SKBMS1 and SKNMC for the recognition by CHM1^319^ CD8^+^ T cells.** ELISpot assay showing count of IFNɣ spots. EwS cells from Figure 8A were co-cultured at indicated effector to target ratios with HLA-A*02:01/CHM1^319^-specific allorestricted TCR transgenic CD8^+^ T cells (CHM1^319^ CD8^+^ T cells) for 24 h. Result from one independent experiment with triplicates is shown. Two-way ANOVA with multiple comparison Tukey´s test was used to calculate p values (B).
